# Tamoxifen enhances stemness and promotes metastasis of ERα36^+^ breast cancer by upregulating ALDH1A1 in cancer cells

**DOI:** 10.1038/cr.2018.15

**Published:** 2018-02-02

**Authors:** Qiang Wang, Jun Jiang, Guoguang Ying, Xiao-Qing Xie, Xia Zhang, Wei Xu, Xuemin Zhang, Erwei Song, Hong Bu, Yi-Fang Ping, Xiao-Hong Yao, Bin Wang, Shilei Xu, Ze-Xuan Yan, Yanhong Tai, Baoquan Hu, Xiaowei Qi, Yan-Xia Wang, Zhi-Cheng He, Yan Wang, Ji Ming Wang, You-Hong Cui, Feng Chen, Kun Meng, Zhaoyi Wang, Xiu-Wu Bian

**Affiliations:** 1 Institute of Pathology and Southwest Cancer Center, Southwest Hospital, Third Military Medical University (Army Medical University), Chongqing 400038, China; 2 Key Laboratory of Tumor Immunopathology, Ministry of Education of China, Chongqing 400038, China; 3 Department of Breast Diseases, Southwest Cancer Center, Southwest Hospital, Third Military Medical University, Chongqing 400038, China; 4 Laboratory of Cancer Cell Biology, Tianjin Cancer Institute, Tianjin Medical University Cancer Institute and Hospital, Tianjin 300060, China; 5 McArdle Laboratory for Cancer Research, University of Wisconsin-Madison, Madison, WI 53706, USA; 6 State Key Laboratory of Proteomics, Institute of Basic Medical Sciences, China National Center of Biomedical Analysis, Beijing 100850, China; 7 Breast Tumor Center, Sun Yat-sen Memorial Hospital, Sun Yat-sen University, Guangzhou 510120, China; 8 Department of Pathology, West China Hospital, Sichuan University, Chengdu 610041, China; 9 Department of Pathology, General Hospital of PLA, Beijing 100853, China; 10 Department of Pathology, No.307 Hospital of PLA, Beijing 100071, China; 11 Laboratory of Molecular Immunoregulation, Cancer and Inflammation Program, Center for Cancer Research, National Cancer Institute, Frederick, MD 21702, USA; 12 Shenogen Pharma Group, Beijing 100085, China; 13 Departments of Medical Microbiology & Immunology, Creighton University Medical School, 2500 California Plaza, Omaha, NE 68178, USA

**Keywords:** breast cancer, estrogen receptor, cancer stem cells, metastasis, endocrine therapy

## Abstract

The 66 kDa estrogen receptor alpha (ERα66) is the main molecular target for endocrine therapy such as tamoxifen treatment. However, many patients develop resistance with unclear mechanisms. In a large cohort study of breast cancer patients who underwent surgery followed by tamoxifen treatment, we demonstrate that ERα36, a variant of ERα66, correlates with poor prognosis. Mechanistically, tamoxifen directly binds and activates ERα36 to enhance the stemness and metastasis of breast cancer cells via transcriptional stimulation of aldehyde dehydrogenase 1A1 (ALDH1A1). Consistently, the tamoxifen-induced stemness and metastasis can be attenuated by either ALDH1 inhibitors or a specific ERα36 antibody. Thus, tamoxifen acts as an agonist on ERα36 in breast cancer cells, which accounts for hormone therapy resistance and metastasis of breast cancer. Our study not only reveals ERα36 as a stratifying marker for endocrine therapy but also provides a promising therapeutic avenue for tamoxifen-resistant breast cancer.

## Introduction

The selective estrogen receptor (ER) modulator tamoxifen has been used as a first-line adjuvant endocrine therapy for hormone-responsive breast cancer for decades^1,2^. Unfortunately, many breast cancer patients developed resistance to tamoxifen therapy associated with cancer metastasis^3,4,5,6^. Multiple mechanisms responsible for endocrine resistance have been proposed, which include deregulation of ER signaling pathway, alterations in signaling that controls cell cycle and survival, and the activation of escape pathways that provide tumors with alternative proliferative and survival stimuli^7,8^. Among the potential mechanistic bases, ERα66 and related signaling pathway are thought to be responsible for the intrinsic or acquired endocrine resistance^7,9^. Tamoxifen resistance of breast cancer cells may be caused by the loss of expression^8^, mutations^9^ or post-translational modifications of ERα66^10^. Until now, ERα66 is the only clinically used biomarker for the selection of tamoxifen therapy^11^. Although survival benefit was observed in patients receiving tamoxifen chemoprevention^12,13,14^, some patients developed tamoxifen resistance and cancer metastasis. However, little is known about the role of ER alpha 36 (ERα36), a truncated variant of ERα66, in tamoxifen resistance of breast cancer.

ERα36 is expressed in breast cancer stem cells (CSCs), which are positive for aldehyde dehydrogenase 1 (ALDH1)^15,16^, but do not express ER66^17,18^. Unlike ERα66, ERα36 lacks transcriptional activation domains (AF-1 and AF-2), but retains dimerization, DNA-binding and partial ligand-binding domains^19^. ERα36 was originally identified as a membrane receptor to mediate the activation of estrogen-related non-genomic signaling pathways, including PI3K/Akt and MAPK/ERK activation^20,21,22^. It also functions in turning on other downstream kinases such as c-Jun N-terminal kinases and mobilizing intracellular Ca^2+^^23,24,25^. Activation of ERα36 contributes to the proliferation and increased invasiveness of breast cancer cells^26,27,28^. Increased levels of ERα36 in breast cancer tissues were associated with reduction in responsiveness to tamoxifen in ERα66^+^ breast cancer patients^29^. Therefore, elevated ERα36 expression in breast cancer may ablate tamoxifen responsiveness.

In this study, we report that tamoxifen promotes breast cancer metastasis via activation of ERα36. Our findings present a novel mechanism by which excessive endocrine treatment leads to patient resistance in association with cancer metastasis. Since ERα36 is widely expressed in human breast cancer specimens, tamoxifen should be excluded from endocrine therapy in ERα36-positive (ERα36^+^) breast cancer patients to reduce tamoxifen-mediated metastasis through ERα36 activation.

## Results

### Increased ERα36 expression correlates with human breast cancer metastasis

**Figure 1:**
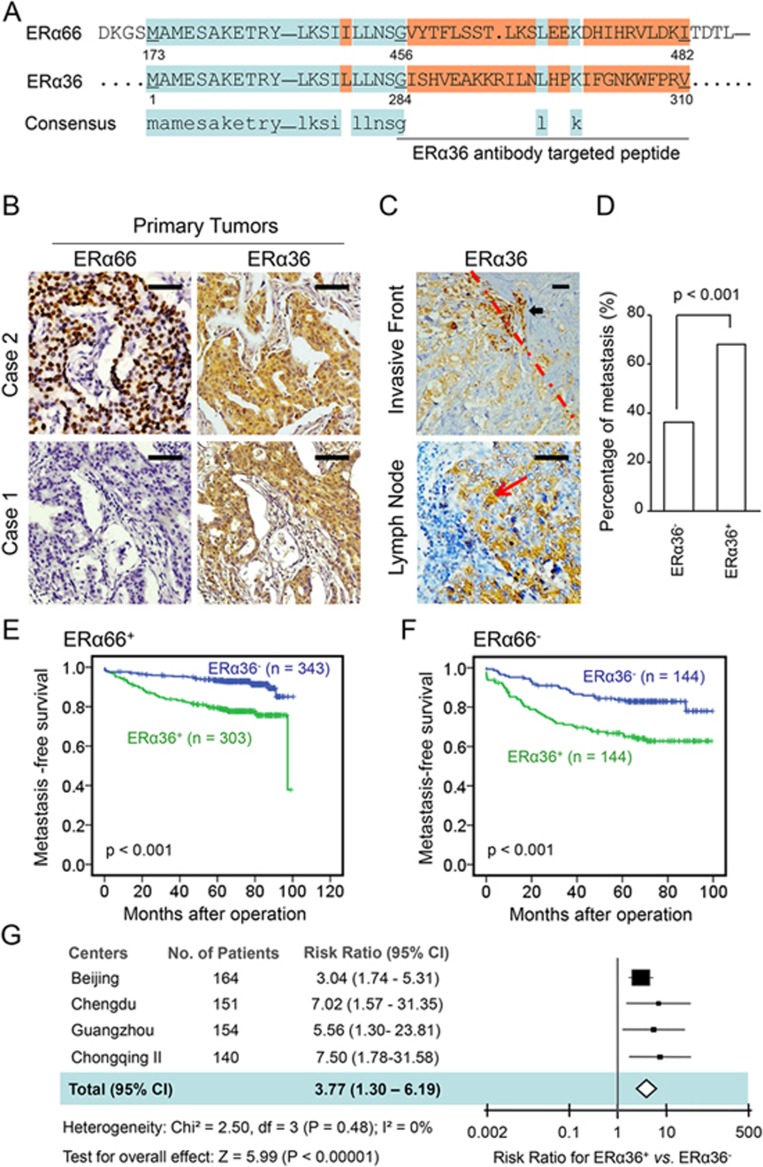
The correlation between high level ERα36 expression in human breast cancer and increased metastasis. **(A)** Generation of a monoclonal antibody-recognizing ERα36. The specificity of the antibody was verified by IHC staining. **(B)** Detection of ERα36 by the monoclonal antibody in primary breast cancer tissues with or without ERα66 expression. Brown staining denotes the immunoreactivity of ERα36 or ERα66. Tumor sections were counterstained by Hematoxylin to label nuclei. Scale bar, 50 μm (Supplementary information, Table S1). **(C)** ERα36 expression (red arrows) in the invasive front (dotted line) of a primary breast cancer and in a metastatic lymph node. Brown staining denotes ERα36 immunoreactivity. Scale bar, 50 μm. **(D)** Higher percentage of lymph node metastases shown by ERα36^+^ breast cancer as compared to ERα36^−^ cancer. Data were analyzed using Pearson's *χ*^2^ test. **(E**, **F)** Kaplan-Meier estimation of metastasis-free survival (MFS) of patients with ERα36^+^ or ERα36^−^ breast cancer in conjunction with ERα66 positivity. *P* value was calculated with two-sided log-rank tests. **(G)** The metastasis hazard ratio of ERα36 expression in breast cancer of independent patient cohorts analyzed with Forest Plot. The size of each square is proportional to the number of patients in each cohort. The area of the squares reflects the study-specific weight. Horizontal lines represent 95% confidence intervals (CI). Diamonds represent the pooled risk ratio and 95% CI of ERα36 expression.

A novel monoclonal antibody was generated against human ERα36 using the C-terminal 27 amino acids of ERα36 as an antigen (Figure 1A). The specificity of the antibody to recognize ERα36 was verified by immunoblotting and immunohistochemical (IHC) staining of breast cancer cells and tissues (Supplementary information, Figure S1A-S1F). The antibody was used to examine the expression of ERα36 in 1 677 human breast cancer samples from five independent cohorts. In the first cohort (Cohort Chongqing) of 1 068 cases, 734 (68.7%) breast cancer specimens were ERα66^+^ and 493 (46.2%) were ERα36^+^ (Supplementary information, Table S1). Among 734 ERα66^+^ samples, 329 (44.8%) co-expressed ERα36. ERα36 was also detected in 164 of 334 (49.1%) ERα66^−^ tumor specimens (Figure 1B). There were two patterns of ER co-expression in breast cancer tissues: ERα36 and ERα66 co-expressed in the same tumor cells or in separate cells (Supplementary information, Figure S1G). The expression level of ERα36 in breast cancer tissues was positively correlated with tumor size (*P* < 0.001), clinical stage (*P* = 0.001), histological grades (*P* < 0.001), lymph node metastasis (*P* < 0.001) and progesterone receptor (PR) expression (*P* = 0.024), but not with patient age (*P* = 0.681), ERα66 (*P* = 0.193) or HER2 (*P* = 0.147) (Supplementary information, Table S1). High levels of ERα36 expression were more frequently detected in the invasive front of tumors and in the metastatic foci of draining lymph nodes (352/423 cases, 83.2%, Figure 1C). Moreover, higher rate of lymph node metastases was detected in patients with higher levels of ERα36 expression in primary tumors (292/429 cases, 68.1%) as compared to patients with lower levels of ERα36 expression (177/487 cases, 36.3%) (Figure 1D). Furthermore, patients with ER36^+^ tumors were more inclined to developing metastasis with lower survival rate, regardless of ERα66 expression (Figure 1E and 1F, Supplementary information, Figure S2A and S2B). These results indicate ERα36 expression in cancer tissues as an independent predictor for increased metastasis and reduced survival of breast cancer patients.

Similar results were obtained in another four independent cohorts of 609 breast cancer cases. These cohorts include the second Chongqing cohort for prospective study, in which patients with ERα36^+^ tumors similarly showed increased rate of metastases (Supplementary information, Table S2). When a substantial effect size was evaluated for pooled cohort data, the hazard ratio for patients with ERα36^+^ versus ERα36^−^ tumors was 3.77 (95% CI, 1.30 to 6.19) (Figure 1G), strongly linking the increased ERα36 expression to metastases of human breast cancer.

### Tamoxifen therapy associates with increased metastasis in ERα36^+^ breast cancer patients

**Figure 2:**
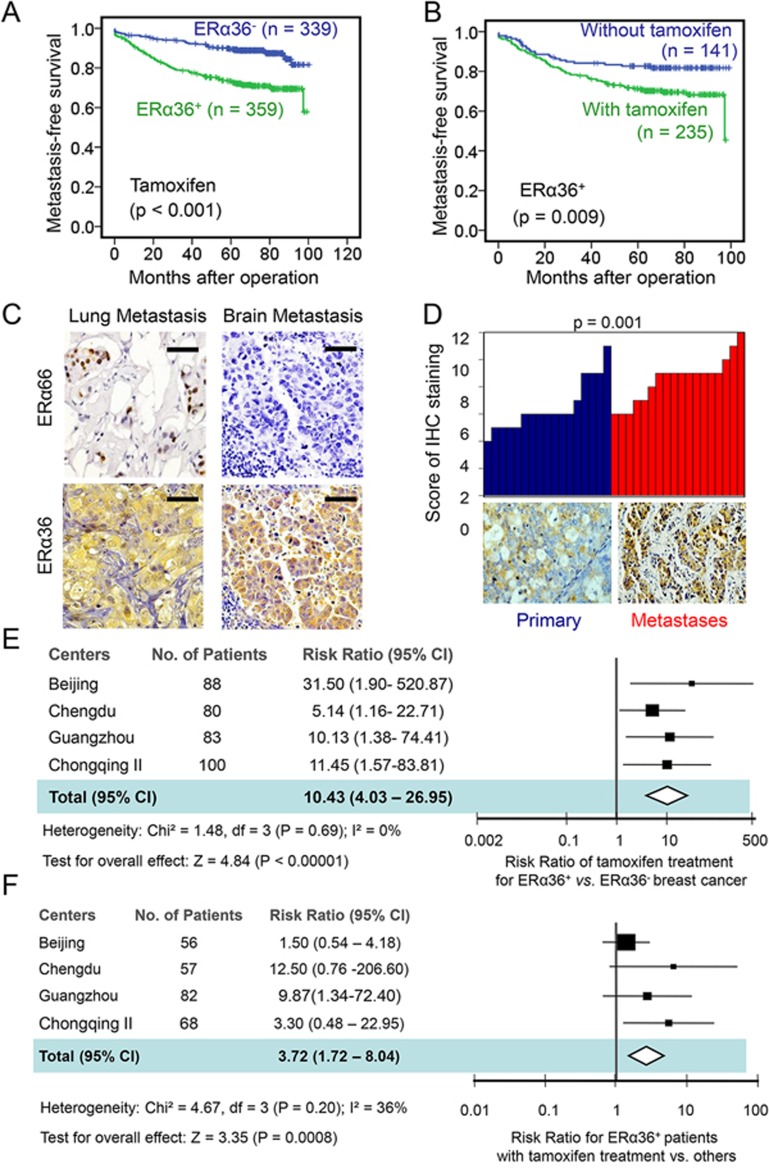
Increased metastasis in tamoxifen-treated patients with ERα36^+^ breast cancer. **(A)** Kaplan-Meier estimation of MFS of tamoxifen-treated patients bearing ERα66^+^ breast cancer with or without ERα36 co-expression. **(B)** MFS of patients with ERα36^+^ breast cancer treated with tamoxifen or other agents. Comparison was made between patient groups with ERα36^+^ (score ≥ 5) and ERα36^−^ (score < 5) cancer. *P* value was obtained from two-sided log-rank tests. **(C)** Immunohistochemical detection of ERα36 in breast cancer of tamoxifen-treated patients with lung and brain metastases with or without ERα66 expression. Scale bars, 50 μm. **(D)** Higher ERα36 expression scores in each metastasis specimens compared to matched primary tumor tissues (*n* = 18). Representative IHC staining for ERα36 was shown with matched primary and metastatic specimens. *P* value was derived from the Mann-Whitney *U* test. **(E)** Forest Plot analysis showing ERα36 expression as a metastasis risk factor for tamoxifen treatment in breast cancer patients. **(F)** Tamoxifen treatment as a metastasis risk factor for patients with ERα36^+^ breast cancers analyzed with Forest Plot. The size of each square in **(E**, **F)** is proportional to the number of patients from respective cohorts. The area of the squares reflects the study-specific weight. Horizontal lines represent 95% CI. Diamonds represent the pooled risk ratio and 95% CI.

We then investigated the relevance of ERα36 to the metastasis of breast cancer following postsurgical endocrine treatment in patients who received tamoxifen and aromatase inhibitors (AIs) in the Cohort Chongqing. There was no difference in the selection of chemotherapy and endocrine therapy for patients with ERα36^+^ or ERα36^−^ tumors (Supplementary information, Table S1). Significantly higher percentage of tamoxifen-resistant specimens were ERα36 positive as compared to treatment naive ones (Supplementary information, Figure S2C). Moreover, higher ERα36 scores were observed in tamoxifen-resistant specimens (Supplementary information, Figure S2D). More importantly, after tamoxifen treatment, patients with ERα36^+^ tumors showed shorter metastasis-free survival (MFS) when compared to those with ERα36^−^ tumors (*P* < 0.001, Figure 2A). In addition, the MFS of patients with ERα36^+^ cancer was significantly shortened if they are treated with tamoxifen (*P* = 0.009, Figure 2B). High levels of ERα36 were detected in metastatic tumor specimens in distant organs from all 18 patients with relapsed diseases after tamoxifen treatment for an average of 2.9 years (ranging from 0.6 to 5.0 years), regardless of the levels of ERα66 expression in primary tumors (Figure 2C). IHC scores of ERα36 were higher in the metastatic lesions than in matched primary tumors (*P* = 0.001, Figure 2D). Multivariate Cox regression analysis revealed negative impact of tamoxifen on disease-free survival (DFS) and MFS of 342 patients whose tumors expressed both ERα36 and ERα66 (DFS: hazard ratio = 5.326; 95%CI, 2.096 to 13.536; *P* <0.001; and MFS: hazard ratio = 4.037; 95%CI, 1.560 to 10.443; *P* = 0.004) (Supplementary information, Table S3). These results indicate that ERα36 is associated with the metastasis potential and poor prognosis of tamoxifen-treated patients with ERα66^+^ breast cancers.

Studies of other four independent cohorts found more metastasis cases in patients with ERα36^+^ tumors than in those with ERα36^−^ tumors after tamoxifen treatment (44/170 versus 4/149, Supplementary information, Table S4). Also, more metastasis cases were identified in ERα36^+^ cancer patients with tamoxifen treatment than in those receiving other treatments (30/113 versus 5/82, Supplementary information, Table S5). Forest Plot analysis of pooled cohort data showed a tamoxifen treatment-response hazard ratio of 10.43 (95% CI, 4.03 to 26.95) for patients with ERα36^+^ versus ERα36 tumors (Figure 2E). The metastasis hazard ratio for tamoxifen treatment versus other therapies in patients with ERα36^+^ tumors was 3.72 (95% CI, 1.72 to 8.04) (Figure 2F). These results confirm the link of ERα36 to the metastasis of breast cancers following tamoxifen treatment.

Unlike tamoxifen, which is an ERα66 antagonist in breast competing with estrogen for binding to the receptor, AIs inhibit estrogen synthesis and thus are commonly used for treating progressive breast cancer in postmenopausal women^30^. In the Cohort Chongqing, chemotherapy did not affect the outcome of patients treated with AIs or tamoxifen (*n* = 244, *P* = 0.816, Supplementary information, Table S6). Regardless of ERα36 and ERα66 expression, there were no differences in overall survival and DFS of postmenopausal breast cancer patients (*n* = 244) treated with tamoxifen or AIs (Supplementary information, Figure S2E and S2F). However, patients with tumors positive for both ERα36 and ERα66 treated with AIs showed longer MFS than those treated with tamoxifen alone (*P* = 0.033, Supplementary information, Figure S2G). No significant difference was found in MFS between patients with ERα36^+^ and ERα36^−^ breast cancer after AI treatment (*P* = 0.151, Supplementary information, Figure S2H). However, Cox Regression analysis revealed that in postmenopausal patients with ERα66^+^/ERα36^+^ tumors, tamoxifen-reduced DFS (HR = 7.705, *P* = 0.008), while AIs improved DFS and MFS (HR= 0.779 and 0.664, Supplementary information, Table S7). These results suggest that tamoxifen therapy promotes tumor metastasis in ERα36^+^ breast cancer patients, whereas treatment with AIs is a favorable factor in the postmenopausal patients with ERα66^+^/ERα36^+^ tumors.

### Increased proliferation and metastasis of breast cancer cells is mediated by tamoxifen-activated ERα36

**Figure 3:**
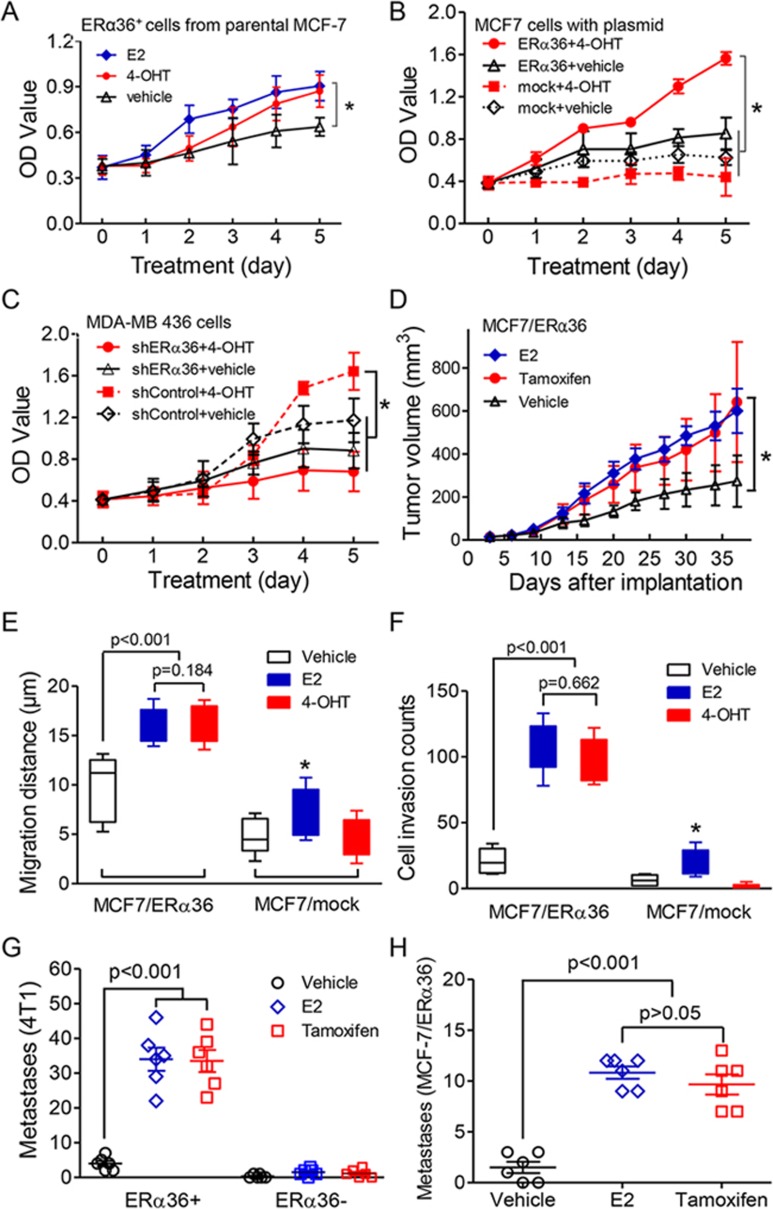
Increased cell viability, invasiveness and metastasis of ERα36^+^ breast cancer cells treated with tamoxifen. **(A)** Assay for cell viability of ERα36^+^ cells sorted from parental MCF-7 breast cancer cells treated with E2 (1 nM) or 4-OHT (1 μM). Ethanol was used as a vehicle control. Each point indicates mean value (± SEM) from three experiments. ^*^P < 0.05. **(B**, **C)** The proliferation of MCF-7/ERα36 and MDA-MB436/shControl cells promoted by 4-OHT. MCF-7/mock and MDA-MB436/shERα36 cells were used as control low ERα36-expressing cells. All cells were treated with 4-OHT (1 μM) for five days and cell number was determined daily. Each point indicates mean (± SEM) of results from three experiments. ^*^P < 0.05. **(D)** Equal rate of growth shown by orthotopically xenografted tumors formed by MCF-7/ERα36 cells after E2 or tamoxifen treatment (*n* = 5 each group). ^*^P < 0.05. **(E)** Elevated invasiveness of MCF-7/ERα36 cells after treatment with E2 (1nM) or 4-OHT (1 μM) in a Transwell assay. Each point indicates mean (± SEM) of results from three experiments. ^*^P < 0.05. **(F)** Increased migration of MCF-7/ERα36 cells observed with E2 (1nM) or 4-OHT (1 μM) treatment. The distance of tumor cells at the leading edge was recorded and measured by Cell Observer. Ethanol was used as a solvent control. ^*^P < 0.05. **(G**, **H)** Pulmonary metastasis of 4T1, mouse breast cancer cell in E2 or tamoxifen-treated animals. Lung metastasis in mice was examined using 4T1-ERα36^+/−^ **(G)** and MCF-7/ERα36 cells **(H)**. Quantitation of metastatic nodules as means ± SEM (*n* = 6 mice/each group). Statistical significance was determined by two-tailed Student's *t* test .

We next examined the effects of tamoxifen on proliferation, tumorigenicity and metastasis of ERα36-expressing breast cancer cells. We enriched ERα36^+^ and ERα36^−^ subpopulations, respectively, from MCF-7 and MDA-MB 436 cell lines (Supplementary information, Figure S3A-C), and found 17β-estradiol (E2) similarly stimulated proliferation of both cell populations (Figure 3A and Supplementary information, Figure S3D). However, 4-hydroxy-tamoxifen (4-OHT), the bioactive metabolite of tamoxifen, promoted the proliferation of sorted ERα36^+^ MCF-7 cells (Figure 3A) but not ERα36^−^ ones (Supplementary information, Figure S3D). Furthermore, we ectopically expressed ERα36 in MCF-7 cell line (MCF-7/ERα36), which constitutively expresses ERα66, or knocked down ERα36 with shRNA from MDA-MB 436 cell line (MDA-MB 436/shERα36), which lacks endogenous ERα66 (Supplementary information, Figures S1A, S3E-S3G). Similar to E2, 4-OHT stimulated the proliferation of both MCF-7/ERα36 cells and MDA-MB 436 cells transfected with control shRNA (MDA-MB 436/shControl) (Figure 3B and 3C and Supplementary information, Figure S4A), but interestingly, 4-OHT lost the enhancement effects on proliferation of MDA-MB 436/shERα36 cells (Figure 3C and Supplementary information, Figure S4B), suggesting that the positive effect of tamoxifen on tumor growth might require ERα36. To further evaluate this effect *in vivo*, either MCF-7/ERα36 or MCF-7/Mock cells were orthotopically transplanted into nude mice, which were subcutaneously inoculated with 0.36 mg 60-day released E2 pellets. Tamoxifen was administered when tumor volume reached ∼200 mm^3^. This treatment promoted growth of MCF-7/ERα36 cells (Figure 3D), whereas it inhibited that of MCF-7/mock cells (Supplementary information, Figure S4C). Therefore, tamoxifen supports growth of breast cancer cells with high levels of ERα36.

*In vitro*, either E2 or 4-OHT treatment significantly enhanced the migration and invasion capacities of ER36-expressing cells, including MCF-7/ERα36 cells and MDA-MB 436/shERα36-ERα36 cells (MDA-MB 436/shERα36 cells that were reintroduced with ERα36) (Figure 3E and 3F, Supplementary information, Figure S4D and S4E). Enriched ERα36^+^ cells from an ERα66-negative mouse breast cancer cell line, 4T1, when transplanted into syngeneic mice, showed significantly increased lung metastasis after tamoxifen or E2 treatment (Figure 3G and Supplementary information, Figure S4F). Similar results were obtained in experiments with sorted MCF-7-ERα36^+^ cells in nude mice (Figure 3H and Supplementary information, Figure S4G), whereas MCF7/mock cells failed to form lung metastasis foci, even with estrogen or tamoxifen treatment. These results suggest the potential of tamoxifen as an ERα36 agonist to promote the proliferation, invasion and metastasis of breast cancer cells.

### ERα36 participates in the maintenance of breast cancer stem cells and co-localizes with ALDH1A1 in human breast cancer tissues

**Figure 4:**
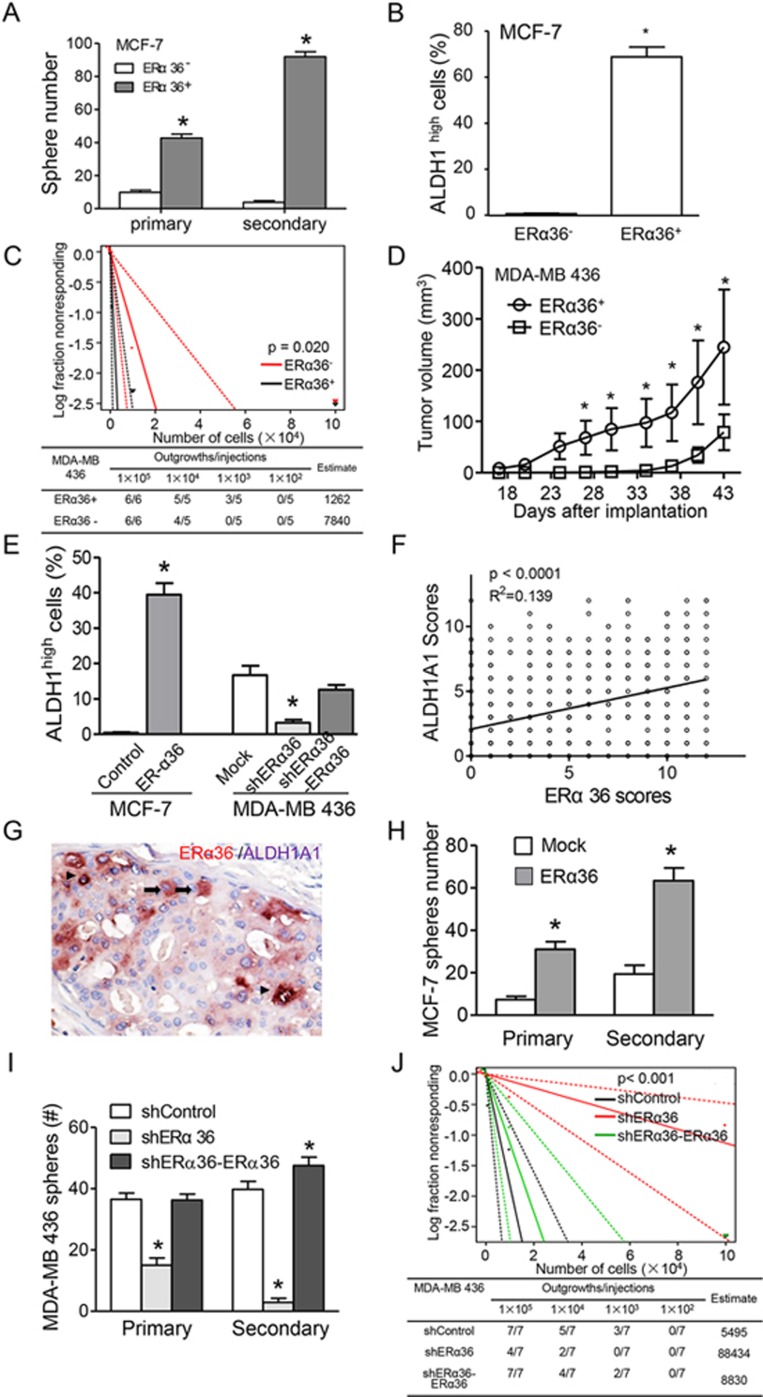
The stemness of ERα36 breast cancer cells and co-distribution of ERα36 with ALDH1A1 in human breast cancer tissues. **(A)** The numbers of mammospheres formed by MCF-7-ERα36^+^ and ERα36^−^ cells at the first and second passage. Columns are mean values (± SEM). *n* = 6. Statistical significance was determined by two-tailed Student's *t* test. ^*^P < 0.01. **(B)** Flow cytometry showing higher percentage of ALDH1^high^ cells in MCF-7- ERα36^+^ cells. *n* = 3. **(C)** Limiting dilution showing higher tumorigenicity of FACS-sorted ERα36^+^ cells of MDA-MB 436 in NOD/SCID mice as compared with ERα36^−^ cells (*n* = 7 each group). Black line refers to ERα36^+^ cells and red line refers to ERα36^−^ cells. **(D)** Increased growth of orthotopical xenograft tumors formed by FACS-sorted MDA-MB 436-ERα36^+^ cells (*n* = 5 in each group). Tumor volume was measured at indicated time points. Data are presented as means ± SEM. Statistical significance was determined by two-tailed Student's *t* test. ^*^P < 0.01. **(E)** Flow cytometry showing higher percentage of ALDH1^high^ cells in ERα36-expressing breast cancer cell variants (MCF-7/ERα36 and MDA-MB 436/shERα36-ERα36) as compared to control cells (MCF-7/mock or MDA-MB 436/shERα36 cells). *n* = 3. **(F)** Positive correlation between the expression of ALDH1A1 and ERα36 in breast cancer specimens analyzed with normal P-P plot of regression stand (dependent variable: ERα36 IHC score). *P* value was calculated with one-way analysis of variance (ANOVA) test. **(G)** ALDH1A1^+^ cancer cells (black arrow) co-expressing ERα36^+^detected by double IHC staining. The arrowheads indicate double expression of ALDH1A1 and ERα36. Brown staining denotes ERα36. Scale bar, 50 μm. **(H**, **I)** Increased primary and secondary generation of mammospheres formed by MCF-7/ERα36 **(H)** and MDA-MB 436/shERα36-ERα36 **(I)** cells as compared to control cells (MCF-7/mock and MDA-MB 436/shERα36). ^*^P < 0.05. **(J)** Limiting dilution showing decreased tumor-initiating capacity of MDA-MB 436/shERα36 cells compared to control cells (MDA-MB 436/shControl and /shERα36-ERα36 cells) in NOD/SCID mice (seven mice in each group).

Since ALDH1A1 plays a pivotal role in the proliferation and metastasis of breast CSCs^31,32^, we investigated the CSC properties of ERα36^+^ cells enriched from MDA-MB 436 and MCF-7 cell lines. *In vitro*, ERα36^+^ cancer cells showed a significantly enhanced capability of mammosphere and colony formation (Figure 4A, Supplementary information, Figure S5A and S5B). In addition, sorted MCF-7-ERα36^+^ cells contained an expanded ALDH1^high^ subpopulation (Figure 4B). Orthotopical tumor formation assays with sorted ERα36^+^ cells from MDA-MB 436 and MCF-7 cell lines (1 ×10^5^ or less) revealed an elevated tumor-initiating ability. Limiting dilution analysis^33^ showed a greater tumorigenic capacity of ERα36^+^ cells than ERα36^−^ ones (Figure 4C and Supplementary information, Figure S5C). Moreover, tumors formed by sorted ERα36^+^ cells from both cell lines grew more rapidly than those formed by ERα36^−^ cells (Figure 4D and Supplementary information, Figure S5D). In addition, the percentage of ALDH1^high^ subpopulation in sorted MCF-7-ERα36^+^ cells was significantly higher than that in their parental cells. Conversely, MDA-MB 436/shERα36 cells exhibited lower percentage of ALDH1^high^ cells than those in MDA-MB 436/mock cells or MDA-MB 436/shERα36-ERα36 cells (Figure 4E, Supplementary information, Figure S5E and S5F). Thus, ERα36^+^ breast cancer cells are more tumorigenic than ERα36^−^ cells, possibly attributing to its co-segregation with CSCs.

We further investigated the association between ALDH1A1 and ERα36 in clinical breast cancer samples. Consistent with previous studies^31^, ALDH1A1 expression was correlated with poor prognosis of breast cancer patients (Supplementary information, Figure S6A and S6B). In cancer specimens, ERα36 levels were positively correlated with ALDH1A1 scores (Figure 4F and Supplementary information, Figure S6C), and higher percentage of ALDH1A1-positive cells was found in ERα36^+^ primary tumors (Supplementary information, Figure S6D). Double IHC staining demonstrated that ALDH1A1-positive breast cancer cells were mostly positive for ERα36, while ERα36 may also be present in ALDH1A1-negative cells (Figure 4G). Moreover, a combination of ALDHA1A1^+^/ERα36^+^ expression suggests a worse prognosis than other patients (Supplementary information, Figure S6E). These findings further support ERα36 as a potential biomarker for breast CSCs.

To test if ERα36 directly promotes cancer stemness, we measured mammosphere formation and tumor-initiating capability upon overexpression or knockdown of *ERα36* in breast cancer cells. We found that *ERα36* overexpression in MCF-7 cells resulted in an increase in mammosphere formation (Figure 4H). In contrast, MDA-MB 436/shERα36 cells showed reduced capacity to form mammospheres during serial passages (Figure 4I and Supplementary information, Figure S6F). *ERα36* knockdown also reduced tumor-initiating capacity of breast cancer cell lines (Figure 4J and Supplementary information, Figure S6G), indicating the capacity of ERα36 to enhance the stem-like property of breast cancer cells.

### Tamoxifen induces the enhancement of stem cell-like properties and ALDH1A1 expression in ERα36^+^ breast cancer cells

**Figure 5:**
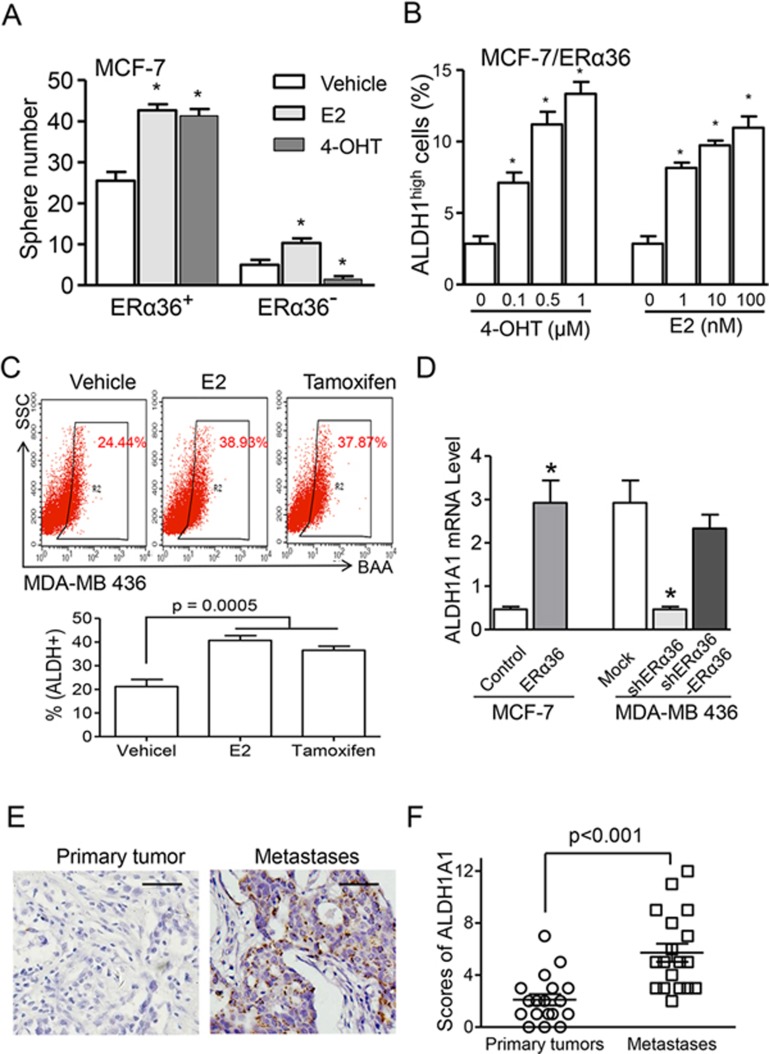
Enhanced stemness of ERα36^+^ breast cancer cells and ALDH1A1 expression after tamoxifen treatment. **(A)** Increased mammosphere formation ability of FACS-sorted MCF-7-ERα36^+^ cells after treatment with E2 or 4-OHT. *n* = 3. **(B)** Increased ALDH1^high^ population in MCF-7/ERα36 cells after E2 and 4-OHT treatment. Cells sorted by FACS were treated with indicated concentrations of 4-OHT or E2. **(C)** Increased ALDH1^high^ population in xenograft tumors formed by MDA-MB 436 cells after E2 or tamoxifen treatment. Flow cytometry was used to assess the percentage of ALDH1^high^ cells in the tumor xenografts. *n* = 4. **(D)** Quantitative real-time RT-PCR analysis showing a positive correlation between *ALDH1A1* mRNA levels and *ERα36* expression in MCF-7 and MDA-MB 436 breast cancer cells. **(E)** IHC staining showing ALDH1A1 in distant metastatic regions from breast cancer patients after tamoxifen treatment. Hematoxylin was used for counterstaining. Scale bars, 50 μm. **(F)** Box plot analysis showing higher ALDH1A1 IHC-scores in metastatic tumors as compared to matched primary tumor from 18 breast cancer patients. *P* value was calculated with Mann-Whitney *U* test. ^*^P < 0.05.

*In vitro*, sorted MCF-7-ERα36^+^ cells showed a substantial capacity of mammosphere formation in the presence of E2 or 4-OHT. In contrast, the mammosphere-forming ability of ERα36^−^ cells was inhibited by 4-OHT (Figure 5A). Treatment with increasing concentrations of 4-OHT also increased the percentage of ALDH1^high^ subpopulation in MCF-7 cells (Figure 5B). Moreover, increased ALDH1^high^ cancer cell population was observed in the xenografted tumors formed by MDA-MB 436 cells (ERα36^+^/ERα66^−^) after treatment with E2 or tamoxifen (Figure 5C).

To explore the signaling capacity of ERα36 in breast cancer cells, gene-expression profiling was performed with sorted ERα36^+^ cells from 4-OHT treated MCF-7 cells. The transcriptome characteristics at the mRNA level in MCF-7 cells with or without ERα36 were analyzed using Affymetrix GeneChip Gene 1.0 ST Transcriptome Array. Totally 1.2% (508/41 000) genes were significantly changed by three times, including 320 genes that were increased to more than tripled, and 188 genes that were reduced to less than a third. The top five up-regulated and down-regulated genes (Supplementary information, Figure S7A) as well as the most differentially regulated signaling pathways (Supplementary information, Figure S7B) were shown. The expression of genes encoding cell adhesion molecules were significantly altered in 4-OHT-treated MCF-7-ERα36^+^ cells, including upregulation of *CLDN14* but downregulation of *ITGB8* (*P* = 0.015. Supplementary information, Figure S7C). Primary ERα36^+^ breast cancer cells expressed a significantly lower level of *CDH1*, but higher level of *SNAI1* in response to 4-OHT treatment, as compared to ERα36^−^ cells (Supplementary information, Figure S7D). Moreover, significantly higher expression of stemness-related genes was observed in 4-OHT-treated primary ERα36^+^ breast cancer cells (Supplementary information, Figure S7E). In addition, *ERα36* mRNA was the only ER transcript highly expressed in an ALDH1^high^ population in MCF-7 cells (Supplementary information, Figure S7F). Interestingly, approximately half of ERα66-targeted genes were upregulated in ERα36^+^ breast cancer cells despite the reduction in ERα66 level, implying the substitution of ERα66 function by ER36.

We next examined the capacity of ERα36 to regulate ALDH1A1 expression in breast cancer cells. ERα36 overexpression in MCF-7 cells (MCF-7/ERα36) resulted in upregulation of *ALDH1A1* mRNA, which was downregulated in MDA-MB 436/shERα36 cells. *ALDH1A1* mRNA level was restored in MDA-MB 436/shERα36 cells re-transfected with ERα36 (MDA-MB 436/shERα36-ERα36) (Figure 5D). Furthermore, in breast cancer patients, ALDH1A1^+^ cancer cells were enriched in metastatic lesions after tamoxifen treatment (Figure 5E and 5F). These results suggest that ERα36 is able to upregulate ALDH1A1 expression in breast cancer cells by responding to the agonist activity of tamoxifen.

### Tamoxifen binding to ERα36 enhances ERα36 nuclear localization and ALDH1 expression in breast cancer cells.

**Figure 6:**
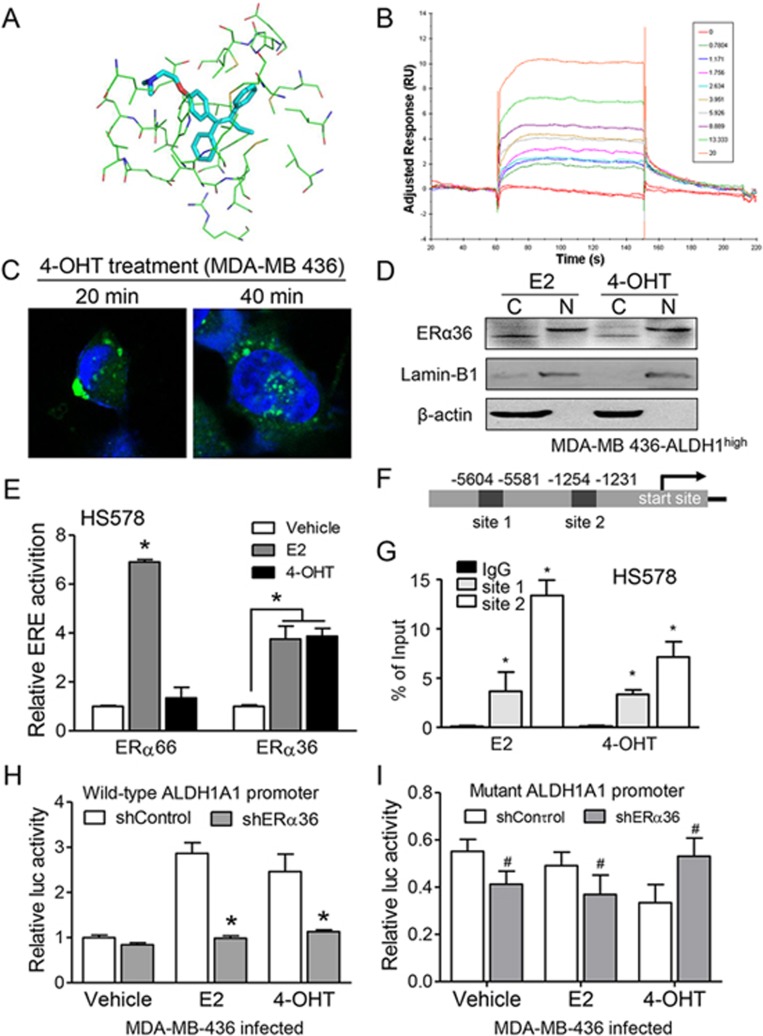
Regulation of *ALDH1A1* expression by tamoxifen-activated ERα36. **(A)** Putative sites in ERα36 involved in interaction with 4-OHT. All aa residues close to 4-OHT in less than 4 Å are shown by lines. The analysis was performed with Discovery Studio 2.0 (Accelrys Software Inc.). **(B)** Binding of 4-OHT to purified GST-ERα36 fusion protein. GST-ERα36 was immobilized to an SPR sensor chip by GST capturing and 4-OHT was introduced as the soluble-phase analyte. The sensorgrams reached equilibrium and rapidly returned to baseline, demonstrating quick interaction kinetics between GST-ERα36 and 4-OHT. The KD was estimated as 11.6 ± 1.0 μM using the Biacore Evaluation Software. **(C)** Nuclear localization of ERα36 (green) in MDA-MB 436 cells after treatment with 4-OHT (1 μM) for 20 and 40 min. Heochst (blue) was used for nuclear staining. Scale bar, 20 μm. **(D)** Western blot of ERα36 in the cytoplasm or nuclei of MDA-MB 436-ALDH1^high^ cells after 4-OHT or E2 treatment. Lamin-B1 was used as a nuclear protein control, β-actin as a cytoplasm protein control. C, cytoplasm; N, nuclei. **(E)** HS578 ERE-luciferase assays showing the transcriptional ability of ERα36 activated by E2 or 4-OHT. ERα36 or ERα66 was transfected into HS578 cells along with an ERE-luciferase element. The transcriptional activity was measured. Error bars represent SEM from mean of triplicate samples. **(F)** Two potential ERE-binding sites in the ALDH1A1 promoter as analyzed by Transcription Element Search System. **(G)** ChIP/PCR analysis of MDA-MB-436 cell lysates showing endogenous ERα36 bound to *ALDH1A1* promoter after treatment with E2 (1 nM) or 4-OHT (1 μM). An unrelated mouse IgG was used as an immunoprecipitation control. ^*^P < 0.05. **(H)** Luciferase activity of the reporter fused to a wild-type *ALDH1A1* promoter observed in MDA-MB-436/shControl cells treated with E2 (1 nM) or 4-OHT (1 μM). DMSO was used as a vehicle control. ^*^P < 0.01. **(I)** Abolished transcriptional activity (shown by relative luc activity) of *ALDH1A1* promoter with mutant ERE sites in MDA-MB 436/shControl cells. Results are presented as mean ± SEM. Statistical significance was determined by two-tailed Student's *t* test. ^#^*P* < 0.05.

We thus further investigated the signaling capacity of ERα36 in breast cancer cells in response to tamoxifen. Despite the reported stimulating activity of estradiol and tamoxifen on ERα36-overexpressing cells^22,34^, direct binding of ERα36 by ligands has yet to be established^35^. Computational modeling and docking analysis revealed that both E2 and 4-OHT fit into the putative ligand-binding domain (aa131-294) in ERα36 that corresponds to aa302-465 in ERα66 (Supplementary information, Figure S8A-S8B). The exposed aa segments in ERα36 are predicted to interact with the hydrophobic groups of 4-OHT and the aromatic rings of aa in ERα36 and 4-OHT may form a π-π interaction (Figure 6A). Ligand-binding assays confirmed that ^3^H-labeled 17-β estradiol (^3^H-E2) bound to ERα36-transfected HS578 cells (Supplementary information, Figure S8C), which was dose-dependently displaced by the unlabeled 4-OHT. ^3^H-E2 also bound to ERα66-transfected cancer cells and the binding was competed by the unlabeled E2 or 4-OHT (Supplementary information, Figure S8D). Surface plasmon resonance (SPR) further demonstrated the capacity of ER ligands to bind purified and immobilized recombinant human ERα36 (Figure 6B and Supplementary information, Figure S8E). Therefore, ER ligands, in particular 4-OHT, directly bind ERα36 through its ligand-binding domain.

To confirm the capacity of ERα36 to regulate ALDH1A1 in breast cancer cells, we visualized ERα36 and found it translocated from the cell membrane and cytoplasm into the nuclei upon 4-OHT treatment of ERα66^−^/ERα36^+^ MDA-MB 436 cells (Figure 6C). ERα36 in MDA-MB 436-ALDH1^high^ cells was detected in the nuclei as well as in the cytoplasm or membrane after treatment with E2 or 4-OHT (Figure 6D). These results indicated a potential transcriptional role of translocated ERα36 in response to either estrogen or tamoxifen, a role similar to that of ERα66^36^. Further studies with HS578 cells carrying estrogen-responsive elements (ERE) luciferase reporter revealed that the ERE reporter was activated by ERα36 after treatment with either E2 or 4-OHT (Figure 6E). Bioinformatics analysis using Transcription Element Search System (http://www.cbil.upenn.edu/cgi-bin/tess) indicated two putative EREs within 5 kb upstream of *ALDH1A1* transcription start site (site 1, bp5581; site 2, bp1231) (Figure 6F). Chromatin immunoprecipitation (ChIP)-qPCR showed that 4-OHT enhanced the binding of ERα36 to two ERE sites in *ALDH1A1* promoter (Figure 6G). Knocking down *ERα36* in MDA-MB 436 cells decreased E2- or 4-OHT-induced transcriptional activity of the *ALDH1A1* promoter (Figure 6H), and this effect of *ERα36* knockdown was abolished when ERE site 2 in *ALDH1A1* promoter was mutated (Figure 6I). Moreover, increased ERE activation in *ALDH1A1* promoter was observed in MDA-MB 436/shERα36 cells after *ERα36* expression was restored and the cells were treated with E2 or 4-OHT (Supplementary information, Figure S8F). These results indicate that E2 or 4-OHT promotes ERα36 binding and activation of EREs in *ALDH1A1* promoter to increase the transcription of *ALDH1A1* in breast cancer cells.

### Targeting ALDH1A1 or ERα36 attenuates tamoxifen-induced breast cancer cell proliferation and metastasis

**Figure 7:**
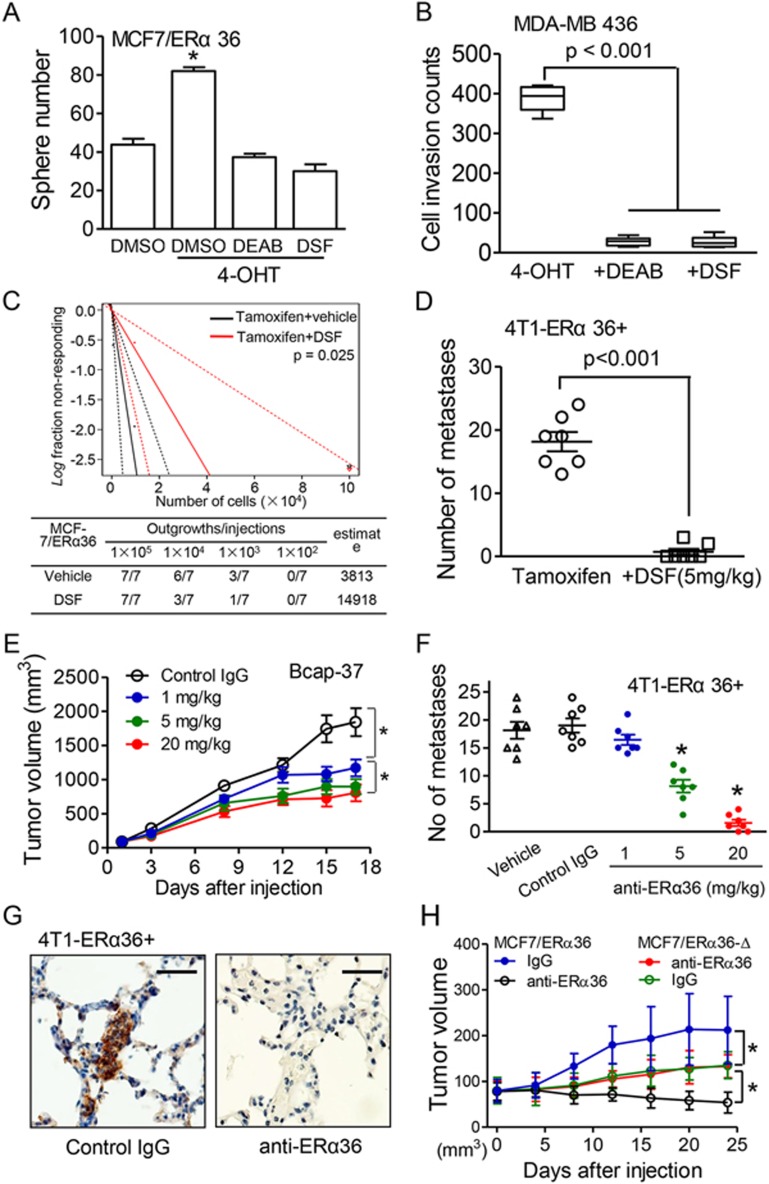
Attenuation of tamoxifen-induced breast cancer proliferation and metastasis by targeting ALDH1A1 or ERα36. **(A)** Reduction of mammosphere formation by MCF7/ER36 cells in the presence of 4-OHT (1 μM) by ALDH1 inhibitors, diethylaminobenzaldehyde (DEAB, 10 nM) or disulfiram (DSF, 0.1 μM). DMSO was used as a vehicle control. Data were presented as mean ± SEM. Statistical significance was determined by two-tailed Student's *t* test. ^*^P < 0.01. **(B)** Attenuation of the invasive ability of MDA-MB 436 cells after 4-OHT treatment (1 μM) in transwell assays by ALDH1 inhibitors DEAB and DSF. *n* = 3. **(C)** Reduction of tumor-initiating frequency of MCF-7/ERα36 cells treated with tamoxifen by DSF. Tumor-initiating frequency was analyzed with limiting dilution of tumor cell transplantation (seven mice each group). **(D)** Reduced number of lung metastases originated from orthotopical xenograft tumors formed by FACS-sorted 4T1-ERα36^+^ cells after DSF treatment (5 mg/kg body weight). Tamoxifen (1 mg/kg body weight), DSF or DMSO was intragastrically administered (ig) every 3 or 4 days for 18 days (arrows). Quantitation is presented as mean ± SEM. Statistical significance was determined by two-tailed Student's *t* test. **(E)** Growth inhibition of xenografted tumors in the presence of tamoxifen by a monoclonal anti-ERα36 antibody. Decreased tumor volume was observed after ERα36 antibody treatment. NOD/SCID mice (7/group) were orthotopically injected with human Bcap-37 cells (1 × 10^6^). When tumor size reached 200 mm^3^, an anti-ERα36 monoclonal antibody (20 mg/kg body weight) was administered through the tail vein every 3 or 4 days. An irrelevant IgG was injected as a control. ^*^P < 0.05. **(F)** Reduction of lung metastases formed by FACS-sorted 4T1-ERα36^+^ cells in the presence of tamoxifen by monoclonal anti-ERα36 antibody. Tamoxifen (1 mg/kg body weight) was intragastrically administrated after orthotopical injection of 4T1-ERα36^+^ cells. Anti-ERα36 monoclonal antibody (20 mg/kg body weight) or control IgG (20 mg/kg body weight) was iv administered through the tail vein every 3 or 4 days for 18 days. ^*^P < 0.05. **(G)** IHC staining of decreased levels of ALDH1A1 in the lung metastatic lesion of breast cancer in nude mice treated with tamoxifen together with monoclonal ERα36 antibody. Hematoxylin was used for counterstaining. Scale bars, 50 μm. **(H)** Failure of anti-ERα36 monoclonal antibody to inhibit the growth of xenograft tumors formed by MCF-7/ERα36-Δ cells (with mutant of aa285-310 in ERα36). The sequence of ERα66 from aa 456 to 481 was used in the mutant ERα36. MCF-7/ERα36 cell-formed xenografts treated with anti-ERα36 antibody or IgG were used as controls. ^*^P < 0.05.

*ALDH1A1* was knocked down in ERα36^+^ breast cancer cells by shRNA to test its potential involvement in tamoxifen-promoted metastasis (Supplementary information, Figure S9A). Reduced mammosphere formation, cell invasion and migration *in vitro* were observed in *ALDH1A1* knockdown breast cancer cells after 4-OHT treatment (Supplementary information, Figure S9B-S9D). In mice, significant reduction in the tumor-initiating ability of MCF-7/ER36 cells with *ALDH1A1* depletion by shRNA was observed (Supplementary information, Figure S9E). ALDH1A1 blockers, diethylaminobenzaldehyde (DEAB)^37^ or disulfiram (DSF)^38^, decreased mammosphere formation of MCF7/ERα36 cells (Figure 7A and Supplementary information, Figure S9F). DEAB or DSF also significantly reduced the migration of 4-OHT-treated MCF-7/ERα36 cells *in vitro* (Figure 7B and Supplementary information, Figure S9G). The tumorigenicity of MCF-7/ERα36 cells promoted by tamoxifen in orthotopic transplantation was attenuated when the mice were treated with DSF (Figure 7C). In addition, DSF also markedly decreased lung metastasis formed by 4T1-ERα36^+^ mouse breast cancer cells with tamoxifen treatment (Figure 7D). These results indicate an important role of ALDH1A1 in breast cancer metastasis mediated by ERα36 activating upon tamoxifen treatment.

**Figure 8:**
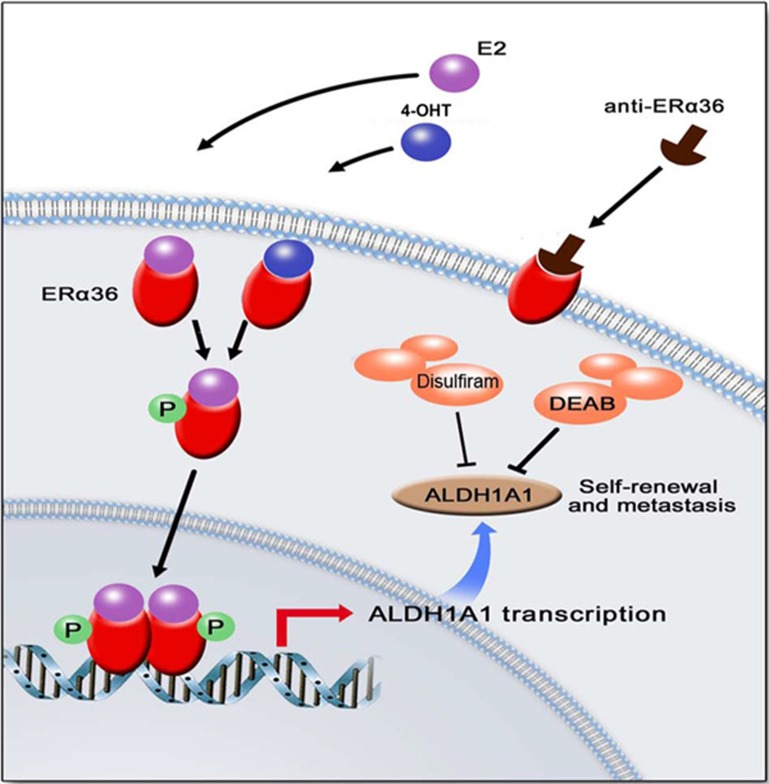
A working model for tamoxifen-ERα36-mediated maintenance of breast CSCs. In ERα36^+^ breast cancer cells, 4-OHT or estrogen induces the nuclear translocation of ERα36 to regulate the transcriptional activity of ERα to increase *ALDH1A1* expression. Elevated ALDH1A1 enriches breast CSCs as source of cancer metastasis. Inhibition of ALDH1 activity by DSF, DEAB or anti-ERα36 antibody eliminates ALDH1^high^ breast CSCs.

Further demonstration of the effect of targeting ERα36 on reduction of breast CSCs and cancer metastasis was shown by the observation that an anti-ERα36 monoclonal antibody potently inhibited the growth of xenograft tumors formed by ERα36^+^ human breast cancer cells in the presence of tamoxifen (Figure 7E and Supplementary information, Figure S9H). Treatment with anti-ERα36 antibody also reduced lung metastasis formed by the mouse ERα36^+^4T1 breast cancer cells in mice (Figure 7F), in association with a considerable reduction in the number of ALDH1A1^+^ breast CSCs in the metastatic foci in the mouse lung (Figure 7G). These results indicate that ERα36 is a potential therapeutic target for preventing breast cancer metastasis promoted by hormone treatment. To further confirm the therapeutic potential of targeting ERα36 in breast cancer cells, we constructed a mutant ERα36 by replacing its C-terminal segment (aa285-310) with a cognate segment of ERα66, for transfection into MCF-7 cells (Supplementary information, Figure S9I). In the xenografts, the anti-ERα36 antibody failed to inhibit the growth of tumors formed by cancer cells expressing the mutant ERα36 (Figure 7H). These results suggest targeting ERα36 is able to reduce the population of ALDH1^high^ CSCs that drive breast cancer development (Figure 8), rendering an opportunity to overcome tamoxifen resistance caused by the ERα36-dependent mechanism.

## Discussion

ERα36 is a truncated variant of the estrogen receptor ERα66, which is expressed by normal breast tissues and breast cancer cells^19,39^. In particular, ERα36 is also expressed in a number of ERα66-negative breast cancer cells such as the MDA-MB 231 cell line as shown in the present study^19,34^. High methylation in ERα66 promoter had little effect on ERα36 transcription^40,41^, indicating no correlation between the expression of ERα36 and ERα66 in breast cancer cells. Since the capacity of ERα36 to bind E2 or tamoxifen remains controversial^35^, our docking model, binding experiments, and SPR assays with purified recombinant ERα36 confirmed the direct interaction between tamoxifen and ERα36. This is in agreement with results showing the capacity of tamoxifen to promote the proliferation of ERα36^+^ breast cancer cells^42^. Thus, the direct interaction of tamoxifen with ERα36 expressed by breast cancer cells, in particular breast CSCs, formed the basis for hormone therapy resistance of the patients who may succumb to tamoxifen-induced cancer metastasis and reduced survival.

Breast cancer cells exhibit remarkable intratumoral heterogeneity^43,44^. Breast CSCs are characterized with self-renewing ability to drive carcinogenesis and progression^45,46^. They are enriched within cell subpopulations with CD44^+^/CD24^−/low^/ESA^+^ surface markers^47^ or enzymatic activity of ALDH1 (ALDH1^high^)^31^. CD44^+^/CD24^−/low^/ESA^+^ and ALDH ^high^ subpopulations represent different subsets of breast CSCs, correlating with different CSC-related bio-behaviors^48,49^. ERα36-positive cells are overlapped with ALDH1^high^ subpopulation, whereas not predominant in CD44^+^/CD24^−/low^/ESA^+^ cells in flow cytometry assays (data not show). Emerging evidence supports that estradiol expands breast CSCs^50,51^. CSC enrichment is also observed in tamoxifen-resistant cell lines and clinical samples^45,52^. Controversially, breast CSCs are responsive to steroid hormone signaling but do not express ERα66^17,18^. Our results here support a cell autonomous pathway for hormonal regulation of stemness via activation of ERα36. Tamoxifen promotes nuclear translocation of ERα36 which could directly regulate transcription of *ALDH1A1*. Therefore, ERα36 functionally replaces ERα66 to mediate genomic hormone signaling in breast CSCs.

The intracellular location of ERα isoforms is important for their function^53^. ERα that localizes on the cell membrane or in the cytoplasm mediates estrogen signaling through non-genomic mechanisms^7^. ERα66 phosphorylation by Erk or Akt serine/threonine kinases is involved in ligand-independent activation^54^. For instance, phosphorylation of ERα66 on Ser305 contributes to tamoxifen resistance^10^. Membrane-bound ERα36 and its non-genomic activities may be involved in *de novo* resistance of breast cancer cells to tamoxifen, in which tamoxifen functions as an agonist of ERα36^55^ to promote the stemness of breast CSCs.

Although the effects of tamoxifen on breast cancer metastasis may also be mediated in a cancer cell extrinsic manner, i.e. via interaction with stromal cells^56^, our current study clearly demonstrated a direct effect of tamoxifen on activation of ERα36 in breast cancer cells to increase their stemness and the metastatic potential. ERα36 enriches breast CSCs to promote cancer metastasis presumably by both genomic and non-genomic mechanisms. We showed that ERα36 transcriptionally enhanced the expression of a key CSC marker ALDH1A1, which is a detoxifying enzyme participating in oxidation of intracellular aldehydes^31,57^. ALDH1A1 has been linked to the self-renewal and metastasis of breast CSCs^58^. This is supported by our observation that ALDH1 inhibitors including DSF attenuate the stemness of breast CSCs. Significant correlation was found between ALDH1A1 expression and the metastasis of human inflammatory breast cancer^59^. A previous report suggested “copper ionophore” activity of ALDH1 inhibitor DSF to generate ROS for its anti-tumor effect. However, such a property of DSF was dependent on the presence of exogenous copper^60^, and the effect of DSF shown in our study was independent of copper, and thus was irrelevant to the purported “copper ionophore” potential.

Our study indicates ERα36 not only as a prognosis biomarker for breast cancer but also as a potential therapeutic target. A previous report suggests that the expression of ERα36 in ERα66^+^ breast cancer is correlated with poor prognosis of patients^29^. Therefore, ERα36 may be utilized to stratify ERα66^+^ breast cancer patients into subgroups that may or may not benefit from tamoxifen therapy. This is based on the clinical results showing that although tamoxifen improves survival expectation of patients in chemoprevention trials^12,14^, tamoxifen administration was also associated with increased metastasis and poor prognosis in a proportion of breast cancer patients. Our investigation of the mechanistic basis resulted in the discovery that tamoxifen activates ERα36 to promote breast cancer cell stemness and metastasis. In clinical specimens, ERα36 expression was strongly correlated with the level of breast CSC marker ALDH1A1, which is regulated by tamoxifen via ERα36. Analysis of patient data (i.e. in Chongqing Cohorts) supports the role of ERα36 in mediating chemoprevention resistance and increased metastasis induced by tamoxifen in ERα36^+^ breast cancer.

Our study also showed that ERα36^+^ breast cancer patients may respond to AIs, consistent with clinical observation that tamoxifen-resistant breast cancer frequently responded to second-line endocrine therapies^2,3^. Postmenstrual patients with ERα36^+^ breast cancer exhibited the worst prognosis after treatment with tamoxifen as compared to AIs, suggesting that blocking estrogen synthesis may be more effective in postmenopausal women^61^. The helix-12 domain in ER66 plays a critical role in protein degradation induced by Fulvestrant^42^. ERα36 lacks the last 4 helixes (helix 9-12) of the ligand-binding domain. Therefore, Fulvestrant appears not able to downregulate ERα36 in breast cancer cells^28^. However, no patient in our cohorts was treated with Fulvestrant, thus the correlation between ERα36 expression in breast cancer and Fulvestant responses has yet to be determined. Nevertheless, our results support the use of AIs as a treatment for patients with ERα36^+^ breast cancer. Based on our study, ERα36 deletion is sufficient to attenuate the proliferation and invasiveness of ERα36^+^ breast cancer cells regardless of the presence of ERα66. It is therefore plausible to develop a personalized therapeutic approach targeting ERα36 in breast cancer.

## Material and Methods

### Breast cancer specimens

This study included five independent cohorts of breast cancer patients who received either radical or modified radical mastectomy. In the Cohort Chongqing, consecutive breast carcinoma tissue samples from 1 068 patients were collected from the years 2006 to 2008. At least 15 axillary lymph nodes from each patient were collected during the operation. Tumor size was determined with the maximum tumor diameter, and lymph node metastasis was histologically diagnosed^62^. The follow-up data were available for 934 of 1 068 patients in the Cohort Chongqing, with a median follow-up time of 67.8 months (ranging from 4.2 to 107.7 months). Four additional cohorts of breast cancer patients with follow-up information were included in this study (609 patients in total). The follow-up data were also available for all patients in four additional independent cohorts, with a median follow-up time of 69.6 months (ranging from 9.8 to 113.8 months) in Cohort Beijing; median follow-up time of 54.3 months (ranging from 18.7 to 69.9 months) in Cohort Chengdu; and 66.7 months (60.5 to 71.7 months) in Cohort Chongqing II; and 56.8 months (42.5 to 87.7 months) in Cohort Guangzhou. The majority of the patients received adjuvant chemotherapy alone (cyclophosphamide, methotrexate, and fluorouracil or anthracycline-based regimen) or combined chemotherapy and endocrine therapy, with or without radiotherapy. Clinical stage was defined according to the WHO classification criteria (2010). Patients with adjuvant endocrine therapy usually received tamoxifen treatment (20 mg/day) for a maximum of 5 years after surgery. The patients were not participants in any clinical trials that used for evaluating the efficiency of other treatments. All breast cancer specimens were collected during operation with written informed consents from the patients and were approved by the institutional review board of each hospital. To make the information of each case accessible to the public, we established a Cancer Research Database (CRD) based on a previously published platform^63^. The basic information, diagnosis, treatment options, clinical follow-up data and the results of immunohistochemistry of all participated patients were deposited in the CRD.

### Generation of a monoclonal anti-ERα36 antibody

An ERα36-derived C-terminal peptide (sequence: GISHVEAKKRILNLHPKIFGNKWFPRV) was synthesized using solid chemistry method and conjugated with KLH for immunization of mice. To purify ERα36 specific antibodies, we selected hybridoma clones that produced an antibody showing the most potent recognition of purified ERα36 ligand-binding domain (LBD) fusion protein. FC-ERα66 LBD fusion protein was used as counter-screening antigen. Antibody production for selected hybridoma clones was performed using roller bottle cell culture. Clarified supernatant was concentrated by ultrafiltration and purified with a Protein A-sepharose column. The purity, concentration and binding ability to the antigen of the antibody were QC tested.

### IHC staining and semi-quantitation

IHC staining for ERα36, ERα66, PR, HER2 (Beijing Golden Bridge Biotechnology Company, Beijing, China) and ALDH1A1 (BD Pharmingen, CA) was performed on primary cancer specimens from 1 677 patients and metastasis in lymph nodes from 423 patients. Hematoxylin and eosin (H&E) staining were performed to ensure cancer tissue and adjacent normal mammary epithelia being on the same section. Tissue slides were deparaffinized with xylene and rehydrated by alcohol gradient. The endogenous peroxidase activity in tissues was blocked with a 0.3% hydrogen peroxide solution and 10% methanol for 30 min at 37 °C. Antigen was retrieved by immersing the slides in 10 mM sodium citrate buffer (pH 6.0) at a sub-boiling temperature for 15 min. The slides were rinsed in phosphate-buffered saline (PBS), then incubated with the primary antibodies overnight at 4 °C in a humidified chamber. The sections were then processed by using Dako Real™ Envison kit (K5007) (Dako North America Inc., CA). Quality assessment was performed on each batch of slides by including a negative control (control IgG replacement of the primary antibody) and a positive control (breast carcinomas known to express high levels of ERα36 protein). A score was assigned to each specimen according to the intensity of the cytoplasmic, nuclear and membrane staining as well as the percentage of positive cells as previously described^10^. The score was assigned to each specimen according to the intensity of the staining on the membrane, cytoplasm and nuclei (no staining = 0; weak staining = 1, moderate staining = 2, strong staining = 3) and the percentage of positive cells (0% = 0, 1%-24% = 1, 25%-49% = 2, 50%-74% = 3, 75%-100% = 4). A final score was obtained by multiplying the intensity score with the percentage score of stained cells, ranging from 0 (the minimum score) to 12 (the maximum score)^64^. The results were analyzed with the receiver-operating characteristic curve (ROC curve, Supplementary Fig. 1D). Scores from 0 to 4 were defined as negative and 5-12 as positive. For ERα66 staining, the scores were assigned according to the intensity and percentage of the cancer cells with nuclear staining. A score of 0 was defined as negative and 1-12 as positive, according to the WHO criteria of IHC for breast cancer^11^.

### Double IHC staining

After tissue slides were prepared, they were incubated with the first antibody goat anti-rabbit HRP polymer (Mach 2 Rabbit HRP-Polymer, Biocare Medical), and Betazoid DAB Chromogen (Biocare Medical) was then used to stain the first marker. The slides were rinsed thoroughly with distilled water, treated with denaturing solution (Biocare Medical) for 3 min to denature anti-rabbit-HRP, and then rinsed for staining with the second antibody. Goat anti-mouse HRP polymer (Mach 2 Mouse HRP-Polymer, Biocare Medical) and Vina Green Chromogen (Biocare Medical) were used for the staining. The slides were finally counterstained with hematoxylin (Biocare Medical).

### Cell culture

Human breast cancer cell lines, MCF-7, MDA-MB 436, Hs578 and Bcap 37 were maintained in Dulbecco's Modified Eagle Medium (DMEM) with 10% FBS, 4T1 mammary tumor cell line was cultured in RPMI-1640 with 10% FBS. Primary tumor cells were cultured in DMEM with 10% FBS. Both media contained 1% penicillin and streptomycin, supplemented with 10% fetal bovine serum (FBS). For 17β-estradiol (E2) and 4-hydroxy-tamoxifen (4-OHT, Sigma Chemical Co. St. Louis, MO) treatment, tumor cells were cultured in phenol red-free DMEM (Hyclone, CA, USA) supplemented with 5% charcoal-filtered FBS (BiochROM AG, Germany).

### *ERα36* transfection and knockdown in breast cancer cells

For stable *ERα36* overexpression or knockdown, lentivirus-expressing *ERα36* (GenBank Accession No. BX640939) or *ERα36*-specific shRNAs^19,25^ were packaged by transfection of HEK293 cells. After 48 h, viruses were harvested and filtered through 0.45 μm filters. Viral titers were determined by using infected 3T3 cells. MOI 5 to 10 were used depending on individual cell lines. Lentivirus and 8 μg/mL polybrene (Invitrogen) were added to the culture of breast cancer cells and incubated overnight at 37 °C in a humidified atmosphere with 5% CO_2_. Gene transduction efficiency was determined by FACS analysis GFP. To eliminate the off-target effects, two shRNAs were designed to target different 3′ UTR region of ERα36. The following shRNAs were used:

Scrambled shRNA: TTCTCCGAACGTGTCACGT

shERα36-1: GCAATTATTCCTTTGCCTTGC;

shERα36-2: GCGTTGCATCATAACATAAGC.

All lentivirus contained GFP-encoding sequences of the infected cells were verified by flow cytometry and immunobloting assays.

### Flow cytometry

Flow cytometry was performed on a FACS Calibur (BD Biosciences, CA). For ERα36 expression, 1 × 10^6^ cultured breast cancer cells or cells from xenograft tumors were incubated with 10 μL of anti-ERα36 antibody at 4 °C for 40 min, followed by incubation with 0.5 μL goat anti-mouse Alexa Fluor 647 antibody (Sparks, MD) at 4 °C for 30 min. The cells were washed with assay buffer for flow cytometry analysis. The experiments of ALDH1 percentage in tumor cells were measured with the The ALDEFLUOR kit (StemCell Technologies, Durham, NC, USA) as previous reports^31^.

### Immunoblotting

Total cell lysates were collected in NP-40 lysis buffer (150 mM NaCl, 1% Nonidet P-40, 50 mM Tris, pH 8.0 and a protease inhibitor cocktail) and protein concentrations were determined by BCA assay (Bio-Rad Laboratories, Inc., Hercules, CA, USA). Equal amounts of proteins from each cell lysate were resolved by SDS-PAGE then transferred to PVDF membranes, which were blocked with 5% skim milk and 0.1% Tween 20 in PBS for 2 h at room temperature. Primary antibodies (ERα36, ERα66 and β-actin) were added overnight at 4 °C, followed by incubation with a secondary antibody at room temperature for 2 h. The antibody against β-actin as a control was from Cell Signaling Technology (#9559).

### Cell viability

Breast cancer cell proliferation was determined with WST-8 kit (Beyotime Inst Biotech, China). Briefly, 2 000 cells/well were seeded into a 96-well flat-bottomed plate. The cells were incubated at 37 °C for 24 h, then were cultured in the presence of different concentrations of E2 or 4-OHT for 5 days. Fresh WST-8 dye was added and the cells were incubated at 37 °C for 2 h before the absorbance was determined by Multiskan Spectrum 1500 (Thermo Scientific, PA) at 450 nm.

### Cell migration

Scratching assays were performed as described previously^65^. Cell migration was recorded with a cell observer (Carl Zeiss Meditec, Germany). The distance of tumor cells at the leading edge was measured with ImageJ.

### Transwell cell invasion assay

Cancer cell migration assays were measured using 8.0 μm pore size Transwell inserts (Costar Corp., Cambridge, MA). Cells were incubated in DMEM with 0.5% FBS overnight and collected with trypsin/EDTA. Washed cells were suspended in serum-free DMEM. DMEM with 10% FBS was placed in the lower chambers of the transwell. Cancer cells (1×10^5^) in 0.1 mL medium were seeded to the upper chamber of the transwell. After 12 h culture at 37 °C, the cells on the upper surface of the membrane were removed. Migrated cells attached to the lower surface of the insert membrane were fixed in 3.7% formaldehyde at room temperature for 30 min, then stained for 20 min with 1% crystal violet and 2% ethanol in 100 mM borate buffer (pH 9.0). For the ALDH1 inhibition, DEAB or DSF was added in the lower chamber of the transwell with indicated concentrations. The number of migrated cells was counted under microscope.

### *In vivo* tumorigenicity and antibody treatment in mice

All mice in the study were handled in accordance to the “Guide for the Care and Use of Laboratory Animals” and the “Principles for the Utilization and Care of Vertebrate Animals”. All animal experiments were approved by the Institutional Animal Care and Use Committee (IACUC) at the Center for Experimental Animals of Third Military Medical University. Ovariectomized female BALB/c nude mice of 6- to 8-week-old (Vital River Laboratories, China) were implanted subcutaneously with 0.36 mg of 60-day release 17-estradiol pellets (Innovative Research, TX), and 1 × 10^6^ cancer cells were injected into the mammary fat pad (with Metrigel) as orthotopical tumor formation models. For the lung metastasis models, mice were placed in a restrainer, and tumor cells were injected orthotopically (4T1-sorted cells) or through the tail vein (MCF-7-infected cells) using a 1-mL syringe^66^. The mice were treated with tamoxifen (Sigma-Aldrich, IL) or DSF for 3 or 4 consecutive days. For antibody treatment, murine ERα36 antibody and control IgG were purified to 95% purity (Sheogen Pharma Group, China). From the day of 4T1-ERα36^+^ cell injection via the tail vein or Bcap 37 cell orthotopical injection into fat pad, tamoxifen at 1 mg/kg body weight, and murine ERα36 antibody or control IgG of 20 mg/kg were iv administered through the tail vein for 3 or 4 consecutive days. Lung metastases were counted histologically with H&E staining of the lungs isolated from mice. Orthotropic tumor growth was monitored every 3 or 4 days using caliper measurements. Tumor volume was calculated by using the formula^67^, tumor volume = 1/2 × larger diameter × (smaller diameter)^2^. Tumors were processed for routine histological examination.

### Mammosphere formation

Mammosphere formation was examined as previously described^65^. FACS-sorted cells were cultured in DF12 medium without phenol red containing b27, EGF and bFGF in ultra-low attachment 24-well plates (Corning, Acton, MA, USA) at the density of 1 000 cells per well. Culture medium and drugs were replenished every 3 days. Mammospheres were counted after 7 days in culture. Experiments were in triplicates. For ALDH1 inhibition, DEAB or DSF was added in the culture medium with the indicated concentration.

### Gene expression microarray

Total RNA was extracted with Qiagen RNeasy Mini Kit (Qiagen). RNA quantity and integrity were analyzed with Nanodrop (Thermo Fisher Scientific, Wilmington, DE) and Nano chip for Eukaryotes on the Agilent 2100 Bioanalyzer (Agilent Technologies, Santa Clara, CA). Gene expression array analysis was performed with Affymetrix GeneChip Human Gene 1.0ST Array system (Affymetrix, Santa Clara, CA). For each sample, 250 ng of total RNA in a volume of 3 μL generate cDNA using Ambion^®^ WT Expression Kit, and fragmentation and labeling with the Affymetrix GeneChip^®^ WT Terminal Labeling Kit (Affymetrix). A total of 5.5 g labeled cDNA, along with GeneChip Hybridization Control reagents, were added into an Affymetrix GeneChip Human Gene 1.0 ST Array. The chips were incubated for 16 h at 45 °C under 60 RPM rotation to allow hybridization. The chips were then washed and stained using GeneChip Hybridization Wash and Stain Kit (Affymetrix) with the Affymetrix GeneChip Fluidics Station 450. Stained arrays were scanned on an Affymetrix GeneChip Scanner 3000 7G.

### Gene set enrichment analysis

Gene set enrichment analysis (GSEA) was performed with normalized data using GSEA v2.0 tool (http://www.broad.mit.edu/gsea/)^68^. We compared the gene expression difference between high and low ERα36-expressing cells from MCF-7. *P* values were analyzed with the Kolmogorov-Smirnov test between the two gene sets.

### Quantitative real time RT-PCR

Total RNA was prepared using TriZol™ reagent (Invitrogen). Five μg of total RNA was subjected to reverse transcription using the PrimeScript RT Master Perfect Real Time Kit (TaKaRa, Japan). A 10 μL volume reaction mix consisted of 1 μL reverse transcription product and 100 nM of each primer. *CDH1, CDH2, SNAI1, SNAI2, TWIST1, POU5F1, SOX2, NANOG, ALDH1A1* primers were used as reported^65,69^. Primers used were shown as follow, ERα-forward: AATTCAGATAATCGACGCCAG, reverse: TTTCAACATTCTCCCTCCTC; ERα46-forward: CATTCTCCGGGACTGCGGTA, reverse: GTACTGGCCAATCTTTCTCTGCC; ERαΔ3-forward: ATGGAATCTGCCAAGAAGACT, reverse: GCGCTTGTGTTTCAACATTCT; ERβ-forward: TAGTGGTCCATCGCCAGTTAT, reverse: GGGAGCCACACTTCACCAT; ERβ-I-forward: CGATGCTTTGGTTTGGGTGAT, reverse: GCCCTCTTTGCTTTTACTGTC; ERβ-II-forward: CGATGCTTTGGTTTGGGTGAT, reverse: CTTTAGGCCACCGAGTTGATT.

### Homology modeling

Homology modeling module of MODELLER was assembled in Discovery Studio 2.0, Accelrys was used. The template structure of ERα66 was obtained from the Protein Data Bank. ERα36 lacks the ligand-binding domain residues aa482-595 but retained aa302-465 of ERα66. To model the structurally conserved core of ERα36, we deleted the corresponding regions from the mutant protein. The structure of 4-OHT was modeled by the sketch tool of Discovery Studio 2.0, Accelrys. The complexes of ERα36 ligand-binding domain and the ligand were built by CDOCKER of Discovery Studio 2.0, Accelrys. The docking procedure was implemented as Chemistry at HARvard Macromolecular Mechanics for configurational exploration with a rapid energy evaluation using grid-based molecular affinity potentials. A rectangular volume was defined around the ER36 ligand-binding cavity which was presumed flexible resulting in 20 best conformations according to the free energy of binding. The conformation of 4-OHT with the maximal free energy binding was selected. The superimposed structure and molecular diagrams of the complexes of 4-OHT and ERα36 were drawn with Profiles-3D.

### Binding assay with ^3^H-labeled 17-β estradiol

Ligand-binding assays were carried out as previously described^70^. Aliquoted Hs578 cells (5 × 10^5^/sample) transfected with ERα36, ERα66 or empty vector were cultured in 24-well plates in DMEM medium containing ^3^H-labeled 17-β-estradiol (72 Ci/mmol) (NET317; NEN) in the presence of different concentration of unlabeled 4-OHT. The cells were incubated at 37 °C for 1 h. The medium was removed and the cells were washed three times with 2% glucose in PBS, then resuspended in 150 μL medium at room temperature. After addition of scintillation fluid, the radioactivity of the cells was measured in a β-counter. Binding of ^3^H-labeled 17-β-estradiol to Hs578 cells containing empty vector was considered as non-specific and the counts were subtracted from the counts obtained with ER-expressing cells.

### ERα36 expression and purification

Human *ERα36* full-length cDNA fused at N-terminal with *GST* and tagged at C-terminal with *Strep*-tag II was expressed in *Escherichia coli* BL21 (DE3) Codon Plus-RIL. Bacteria were cultured in LB medium at an OD^600^ of 0.5 with 0.1 mM IPTG, and grown for 5 h at 28 °C. Bacteria were harvested by centrifugation, resuspended in Buffer Sol-ER (50 mM HEPES pH 7.9, 180 mM NaCl, 5 mM KCl, 1 mM EDTA, 5% Glycerol, 0.05% Triton, 1 mM DTT) plus 1 mM PMSF and lysed by sonication after 1 mg/mL lysozyme treatment. After centrifugation at 13 000× *g* for 30 min at 4 °C, the soluble extracts were applied to a StrepTrap HP-column (GE Healthcare) equilibrated with Buffer Sol-ER. The column was then washed extensively with Sol-ER and the bound protein was eluted by 2.5 mM desthiobiotin. The column purified protein was concentrated by Amicon Ultra-4 10K centrifugal filter (Merck Millipore) and dialysed in D-Tube Dialyzer Mini (Novagen) overnight at 4 °C against Sol-ER. The chromatograph was run on an AKTA Explorer system (GE Healthcare) and the purified protein was shown as a major band of GST-ERα36 by Coomassie brilliant blue staining. A lower minor band was characterized as a premature N-terminal fragment of ERα36.

### Surface plasmon resonance

The binding of 4-OHT to purified recombinant ERα36 was measured by SPR on a Biacore T200 optical biosensor (GE Healthcare). A GST capturing kit from GE was used for research-grade Series S CM5 chip surface preparation. Following standard protocols provided by the manufacturer, the anti-GST antibody (GE) was first immobilized on the chip surface via amine coupling to the free carboxyl groups on the chip surface using standard NHS/EDC procedures with PBS-P+ (20 mM phosphate buffer with 2.7 mM KCl, 137 mM NaCl and 0.05% Surfactant P20, pH 7.4) as the Running Buffer. Approximately 12 000 response units (RU) of anti-GST antibody were immobilized to each of the two flow cells (Fc1 and Fc2). Purified GST and GST-ERα36 fusion proteins were then captured by the anti-GST antibody in Fc1 and Fc2, respectively. Due to the difference of the molecular weight, about 1 000 RU of the GST protein in Fc1 and 2 500 RU of the GST-ERα36 fusion protein in Fc2 were captured. All data were background adjusted in real time (Fc2-Fc1). Immediately before analysis, 1 mM 4OHT (sigma) stock solution in DMSO was diluted in Running Buffer to yield a series concentration of working analytes while maintaining DMSO at 2%. The analytes were injected at a low rate of 30 μL/min over Fc1 and Fc2 and allowed to associate with the proteins for 90 s and dissociate for 60 s. Data analysis of affinity with Ymax as Rmax was performed using the Biacore Evaluation Software.

### Immunofluorescence

FACS sorted ALDH1^high^ breast cancer cells from MDA-MB 436 were seeded on coverslips in 24-well plates overnight, then were treated with 4-OHT for 20 or 40 min. For the detection of lung metastases in mice, specimens were embedded in Tissue-Tek OCT (optimal cutting temperature) compound at −20 °C for cryostat sections (6 μm), which were mounted on poly-l-lysine-coated coverslips and fixed in acetone for 20 min at 4 °C. The samples were fixed in 4% paraformaldehyde for 20 min at room temperature. After blocking and permeabilizing with preimmune goat serum for 30 min at 37 °C, the samples were incubated with ERα36 antibody (1:300) overnight at 4 °C. The cells were washed with PBST and incubated with goat anti-mouse IG antibodies conjugated with Cy3 or FITC (Abcam, USA) for 30 min at 37 °C. The number of positive cells in at least 10 randomly selected microscopic fields was normalized to the total number identified by counterstaining with Heochst 33 258 to detect the nuclei. Samples were observed using laser confocal scanning microscope (Leica TCS-SP5, Germany).

### Chromatin immunoprecipitation-PCR

MCF-7 cells were fixed with 1% formaldehyde for 15 min, and sonicated to obtain 1.5-kb to 500-bp chromatin fragments. The chromatin was diluted and incubated with antibodies overnight followed by 2 h incubation with salmon sperm DNA-preblocked protein A-Sepharose to precipitate antibody-bound chromatin. After stringent washing, immunoprecipitated complexes were eluted for reversal of crosslinking and DNA purification. Specific antibody-enriched samples were analyzed by PCR or quantitative PCR to amplify selected genome loci. A 1% input serves as a control. The primer sequences used for PCR of ALDH1A1 promoter are:

Site 1-forward: 5′- CATTGCATCCACACATGGC-3′

Site 1-reverse: 5- GGGAACACAGAGCCAAATC-3′.

Site 2-forward: 5- CTCTTGTGGAGAATAGGGTAG-3′

Site 2-reverse: 5- GACATACAGAGGGTGAGTAGC-3′.

Control forward: 5′- ATGAGTAAAAGCTTCCGGAGG-3′

Control reverse: 5′- TGGCTCATGTTTCTGTAGGC-3′.

### Luciferase reporter assays

Hs578 cells were transfected in batches in 48-well plates using 2.5 ng ERα36 or ERα66 and 50 ng tk-ERE-luc vector per well, as well as ALDH1A1 wild-type/mutant promoter-Luc for 48 h. The cells were then incubated with indicated ER ligands for 24 h. The cells were then lysed, and firefly luciferase emission was detected upon addition of firefly luciferase substrate (Promega) on a PerkinElmer Victor 3-V plate reader. β-gal was analyzed using the Tropix β-gal actosidase detection kit (Tropix), and emission was detected on a PerkinElmer Victor 3-V plate reader. Luciferase counts were normalized to β-gal counts obtained in each well.

### Construction of mutant ERα36 plasmids and transfection

HA-tagged deletion mutant of ERα36-Δ (ERα36-Δ) were generated using a Quik-Change Mutagenesis Kit (Strategene). Primers are:

ERα36-Δ forward: 5′-GCGAATTCACCATGGCTATGGAATCTGCCAAG-3′

ERα36-Δ Reverse: 5′-GCGGGATCCCTGTGATCTTGTCCAGGACTC-3′

All plasmids were verified by restriction enzyme digestion and DNA sequencing. Plasmids transfection was performed as previous report^10^. The ShRNA plasmid for ALDH1A1 was purchased from Genechem CO. LTD (Shanghai, China).

### Statistical analyses

Statistical analyses were performed with SPSS 19.0 (SPSS Inc, Chicago, IL, USA), Graphpad Prism 5 (GraphPad Software, Inc., La Jolla, USA), Review Manager 5.0.16 (The Nordic Cochrane Centre, Copenhagen, Denmark), and GSEA v2.0 tool (Cambridge, MA). Fisher's exact test was used to assess the association between ERα36 expression and clinico-pathological characteristics. Cox proportional-hazards regression model was used for subsequent multivariate analyses of factors with prognostic significance. Kaplan-Meier estimates followed by log-rank test were used for univariate analyses of cumulative tumor recurrence and metastasis. ROC analysis^71^ was used to determine the optimal cut-off point. The correlation between ERα36 expression and clinical parameters was analyzed by Spearman rank correlation. Survival rates were estimated by Kaplan-Meier analysis. Comparisons between different subgroups of patients were conducted with a log-rank test. Estimation of hazard ratios was presented with 95% confidence intervals. The differences in the expression levels of genes were analyzed with Mann-Whitney *U* test. All statistical tests were two-sided, and *P* ≤ 0.05 was considered statistically significant.

## Author Contributions

QW, JJ, GY and X-WB designed the experiments, X-QX, ES, HB, XMZ, YT, X-HY and Y-FP collected human samples and constructed database, QW, BW, Z-XY, Y-XW, SX and Z-CH performed the experiments, BH, XQ and JJ followed up the patients, QW, XZ, JJ, Y-HC, FC, KM, ZW and X-WB analyzed the results, QW, XZ, WX, YW, JMW, Y-HC, ZW and X-WB wrote the manuscript. X-WB supervised the study.

## Competing Financial Interests

The authors declare no competing financial interests.

## Supplementary Information

Supplementary information, Figure S1 Validation of the specificity of the monoclonal antibody against human ERα36.

Supplementary information, Figure S2 The association of ERα36^+^ expression with the poor prognosis of human breast cancer patients treated with endocrine therapies.

Supplementary information, Figure S3 Establishment of cell lines by lentivirus delivery of gene construct and selection.

Supplementary information, Figure S4 Enhanced proliferation and lung metastasis of ERα36^+^ cells resulted from E2 or tamoxifen treatment.

Supplementary information, Figure S5 ERα36 maintains breast cancer cell stemness

Supplementary information, Figure S6 Association of increased ALDH1A1 expression with poor prognosis in breast cancer patients.

Supplementary information, Figure S7 Enhanced stemness of ERa36^+^ cells by tamoxifen treatment.

Supplementary information, Figure S8 Requirement of ERα36 for the induction of ALDH1A1 expression in breast cancer cells treated with estrogen or tamoxifen

Supplementary information, Figure S9 Reduced self-renewal ability of ERα36^+^ cells by *ALDH1A1* knockdown

Supplementary information, Table S1 The Correlation between ERα36 Expression and ClinicopathologicalCharacteristics in Breast Cancer Patients from Cohort Chongqing (n=1 068 cases)

Supplementary information, Table S2 Prognosis of Patients with ERα36^+^ or ERα36-Breast Cancer in Four Independent Cohorts (n=609)

Supplementary information, Table S3 Multivariate Analyses of Disease-Free Survival (DFS) and Metastases-Free Survival (MSF) in 342 Patients with Breast Cancer Positive for Both ERα36 and ERα66

Supplementary information, Table S4 Responses of Tamoxifen Treatment to Patients with ERα36^+^ or ERα36^−^ Breast Cancer in Four Independent Cohorts

Supplementary information, Table S5 Responses of Patients with ERα36^+^ Breast Cancer to Tamoxifen or Others in Four Cohorts

Supplementary information, Table S6 Postmenopausal Breast Cancer Patient Groups Treated with Aromatase Inhibitors (AIs) and/or Tamoxifen with Tumors Expressing both ERα36 and ERα66 (n=244)

Supplementary information, Table S7 Multivariate Analyses of Disease-Free Survival (DSF) and Metastasis-Free Survival (MSF) of Postmenopausal Patients with ERα36^+^/ERα66^+^ breast cancer
