# Supplementary material for: Tamoxifen enhances stemness and promotes metastasis of ERα36+ breast cancer by upregulating ALDH1A1 in cancer cells
Source: Cell Res. 2018 Feb 2;28(3):336–58. doi: 10.1038/cr.2018.15 (PMC5835774; doi:10.1038/cr.2018.15)
Supplement: Supplementary information, Figure S1 — Validation of the specificity of the monoclonal antibody against human ERα36. [file cr201815x1.pdf]

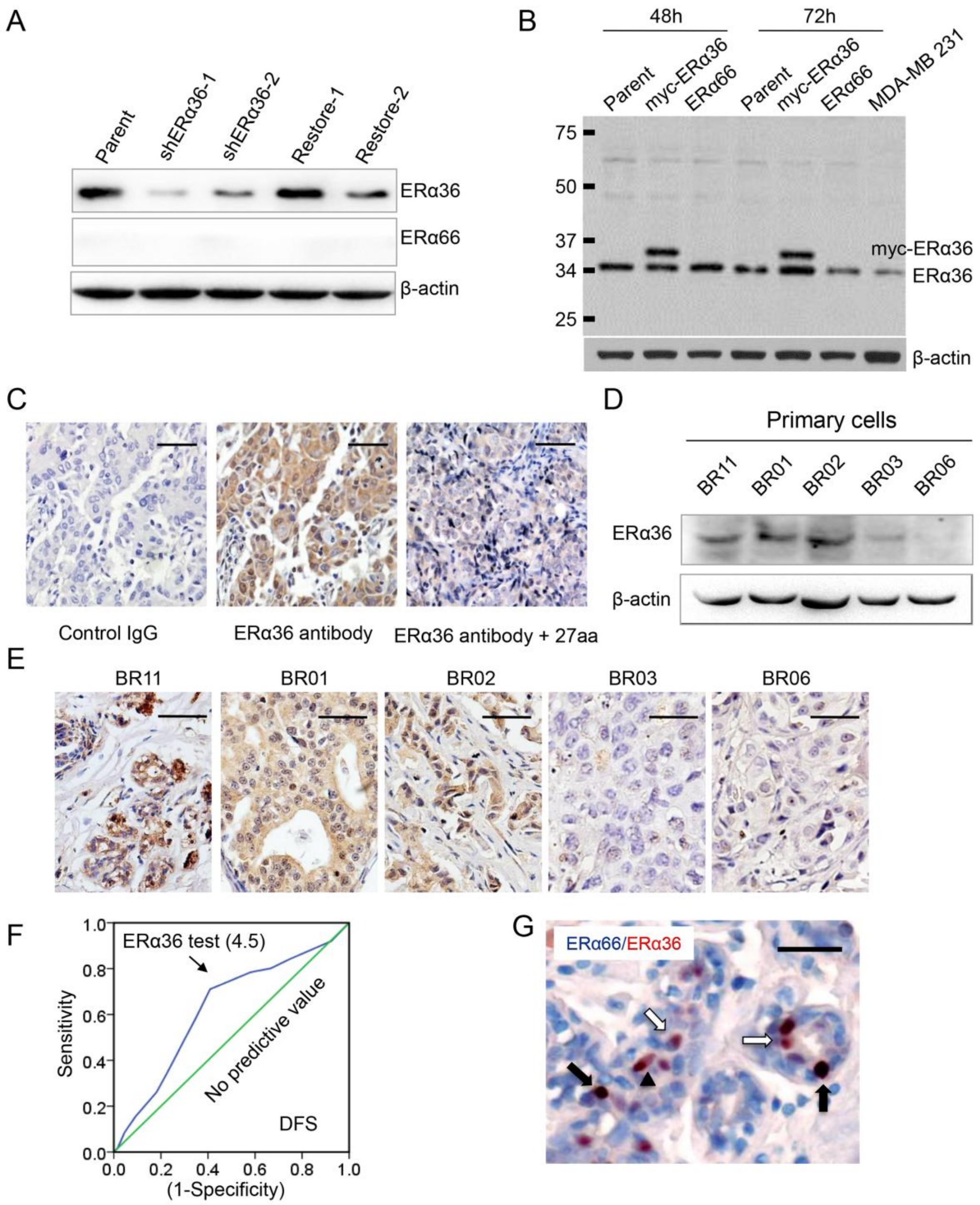

Wang Q, *et al*. Figure S1

Figure S1. Validation of the specificity of the monoclonal antibody against human ERα36.

A. The specificity of ERα36 antibody confirmed by immunoblotting with MDA-MB 436 cells transfected with shERα36. No ERα66 expression was detectable in parental and ERα36 knockdown cells. Beta-actin was used as a loading control.

B. Immunoblotting performed with HEK293 cells transfected with myc-tagged

ERα36 or wild type ERα66 for 48 and 72 hours. No immunoreactivity of the ERα36 antibody with ERα66 protein was detected. ERα36 expression was found in parent HEK293 cells. MDA-MB 231 cells were used as a positive control.

C. Immunohistochemical staining performed to validate the specificity of the ERα36 antibody. Human breast cancer specimens were stained with IgG or the ERα36 antibody with or without the peptide containing specific C-terminal 27 amino acids of human ERα36. Brown staining denotes ERα36 immunoreactivity. Hematoxylin was used for counterstaining. Scale bar = 50 μm.

D. Representative results of immunoblotting of ERα36 in primary breast cancer cells cultured from the breast cancer tissue. ERα36 expression was detected in the cells from cases BR11, BR01 and BR02, which is consistent with the results of IHC staining.

E. Representative results of IHC staining for ERα36 performed on primary breast cancer samples. ERα36 expression was observed in primary human breast cancer samples (case numbers BR11, BR01 and BR02). Scale bar = 50 μm.

F. Receiver-operating characteristic curve used to determine the cut-off score for the level of ERα36 expression. Score ≥ 5 was defined as ERα36^{+} and ≤ 4 was ERα36^{-}. The sensitivity and specificity for each clinical sample were plotted.

G. Double IHC staining of ERα66 (black arrow) and ERα36 (white arrow) showing the co-expression in breast cancer specimens. Black arrowhead shows double expression of ERα66 and ERα36. Tumor sections were counterstained with hematoxylin. Scale bar = 50 μm.
