# Supplementary material for: Tamoxifen enhances stemness and promotes metastasis of ERα36+ breast cancer by upregulating ALDH1A1 in cancer cells
Source: Cell Res. 2018 Feb 2;28(3):336–58. doi: 10.1038/cr.2018.15 (PMC5835774; doi:10.1038/cr.2018.15)
Supplement: Supplementary information, Figure S2 — The association of ERα36+ expression with the poor prognosis of human breast cancer patients treated with endocrine therapies. [file cr201815x2.pdf]

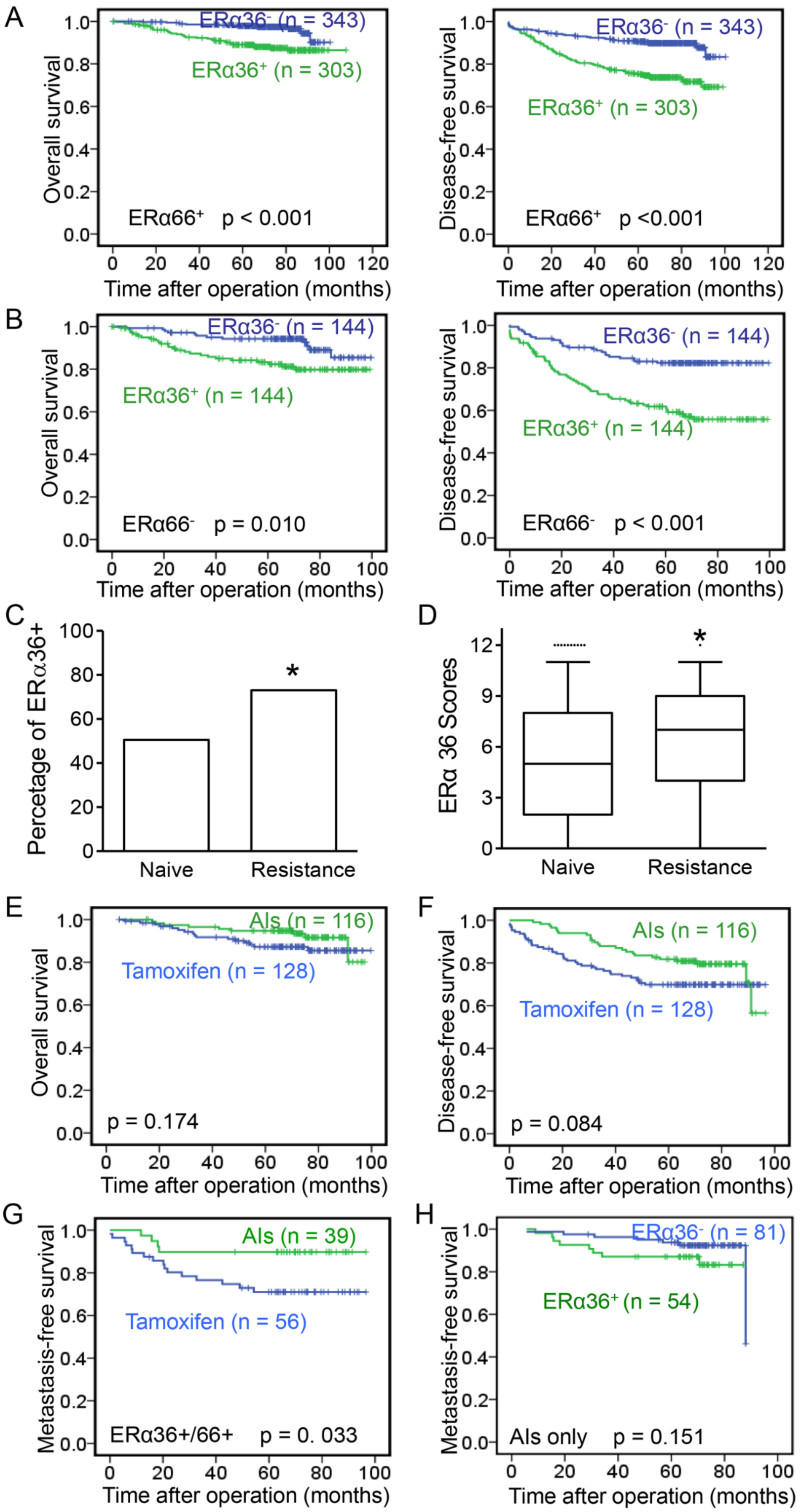

Wang Q, *et al*. Figure S2

Figure S2. The association of ERα36$^+$ expression with the poor prognosis of human breast cancer patients treated with endocrine therapies.

A and B. Kaplan-Meier estimation of overall survival (OS, A) and disease-free survival (DFS, B) of patients with ERα36$^+$ or ERα36$^-$ breast cancer in conjunction with ERα66 positivity. Worse prognosis was observed in patients with ERα36$^+$ breast cancer than those with ERα36$^-$ in each group.

C. Significantly higher percentage of ERα36 positive was observed in tamoxifen-resistant specimens than treatment na ïve ones. * $p < 0.001$.

D. Higher ERα36 scores of specimens of tamoxifen-resistant patients were observed than treatment na ïve ones. * $p = 0.0013$.

E and F. Kaplan-Meier estimation of OS (E) and DFS (F) in postmenopause patients with ERα66$^+$ breast cancer treated with tamoxifen and AIs. No significant difference was observed.

G. Kaplan-Meier estimation of DFS of postmenopause patients with ERα66$^+$/ERα36$^+$ breast cancer treated with AIs or tamoxifen. Worse prognosis was observed in patients receiving tamoxifen treatment.

H. Kaplan-Meier estimation of MFS of patients with ERα36$^+$ or ERα36$^-$ breast cancer receiving AIs treatment only. No significant difference was detected.
