# Supplementary material for: Tamoxifen enhances stemness and promotes metastasis of ERα36+ breast cancer by upregulating ALDH1A1 in cancer cells
Source: Cell Res. 2018 Feb 2;28(3):336–58. doi: 10.1038/cr.2018.15 (PMC5835774; doi:10.1038/cr.2018.15)
Supplement: Supplementary information, Figure S3 — Establishment of cell lines by lentivirus delivery of gene construct and selection. [file cr201815x3.pdf]

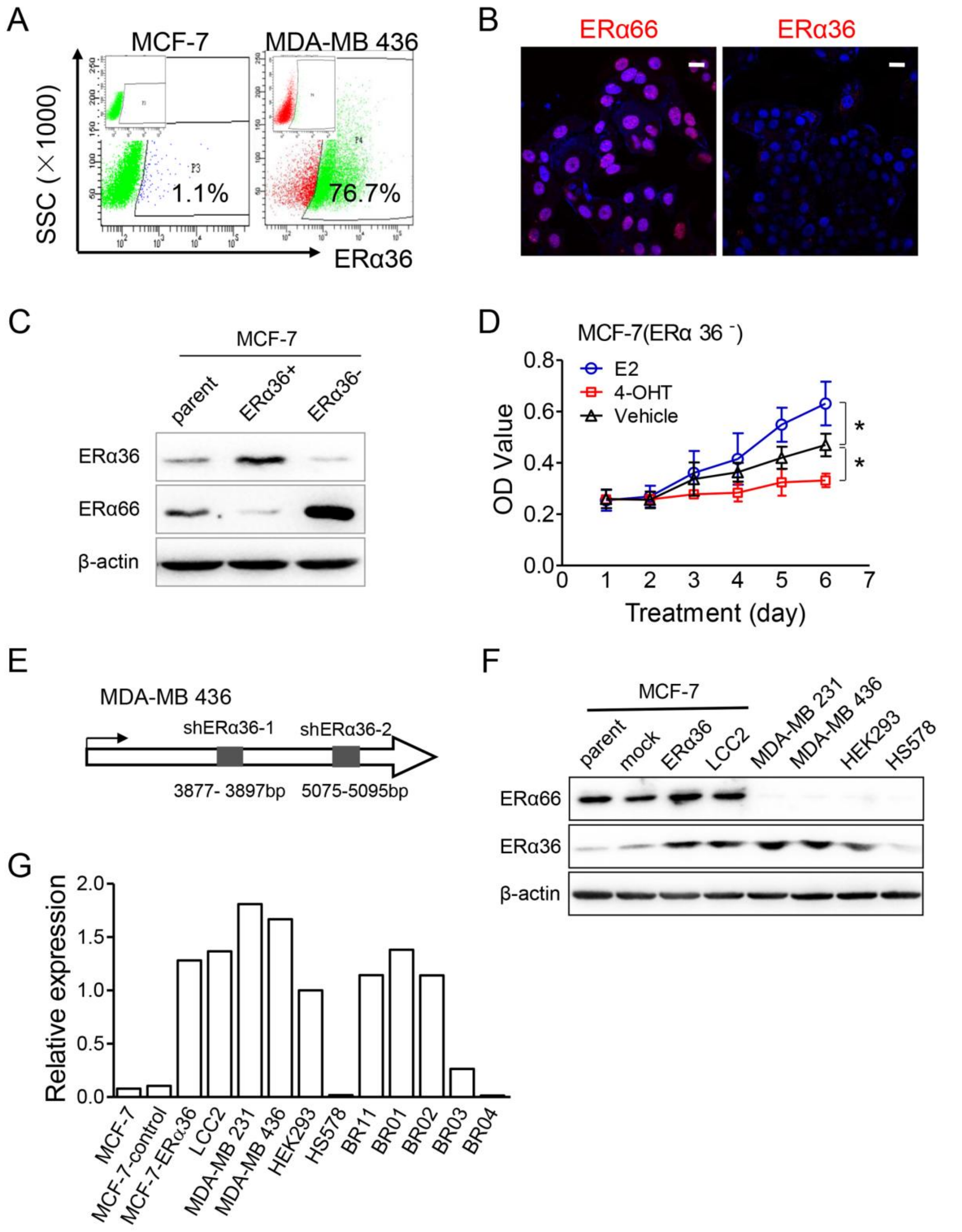

Wang Q, *et al*. Figure.S3

Figure S3. Establishment of cell lines by lentivirus delivery of gene construct and selection.

A. ERα36$^{+/-}$ cells in MCF-7 and MDA-MB 436 cell lines sorted utilizing flow cytometry. Higher percentage of ERα36^{+} cells was detected in MDA-MB 436 than in

MCF-7 cells.

B. ERα66^{+} MCF-7 cells expressing low level of ERα36 with confocal microscopy. Hoechest was used to localize the nuclei. Bar = 20 μm.

C. Immunoblotting of ERα36 and ERα66 performed with FACS sorted ERα36$^{+/-}$ cells from MCF-7 cell line. Lower levels of ERα66 expression was detected in ERα36^{+} than in ERα36^{-} cells. Beta-actin was used as a control.

D. The proliferation of FACS sorted MCF-7-ERα36^{-} cells was promoted by E2 (1 nM), but inhibited by 4-OHT (1 μM). Ethanol was used as vehicle controls. Each point indicates the mean value (± SEM) from three experiments. * $P < 0.05$.

E. ERα36 shRNAs targeting at the sequence in 3' UTR of ERα36. The shRNA transduction efficiency in MDA-MB 436 cells was confirmed by immunoblotting as Supplementary Fig. 1A.

F. Immunoblotting of ERα36 and ERα66 with whole lysates from MCF-7/mock or MCF-7/ERα36 cells, LCC2, MDA-MB231, MDA-MB436, HEK293 and HS578 cells.

G. Expression levels of ERα36 mRNA in MCF-7/ERα36 and ERα36^{+} cell lines, as well as primary cells. Beta-actin was used as a control.
