# Supplementary material for: Tamoxifen enhances stemness and promotes metastasis of ERα36+ breast cancer by upregulating ALDH1A1 in cancer cells
Source: Cell Res. 2018 Feb 2;28(3):336–58. doi: 10.1038/cr.2018.15 (PMC5835774; doi:10.1038/cr.2018.15)
Supplement: Supplementary information, Figure S4 — Enhanced proliferation and lung metastasis of ERα36+ cells resulted from E2 or tamoxifen treatment. [file cr201815x4.pdf]

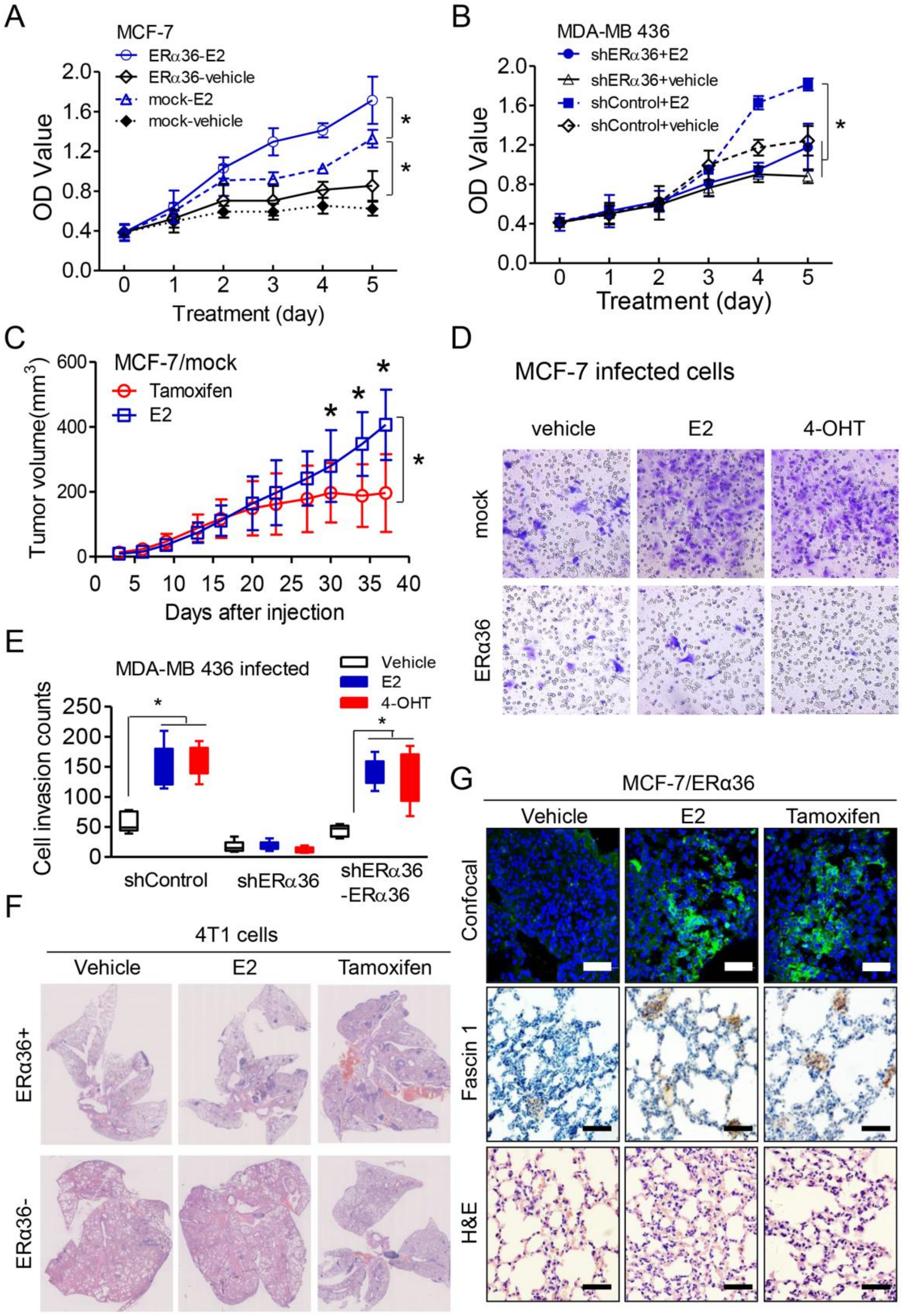

Wang Q, *et al*. Figure S4

Figure S4. Enhanced proliferation and lung metastasis of ERα36^{+} cells resulted from E2 or tamoxifen treatment.

A and B. The proliferation of MCF-7/mock or MCF-7/ERα36 and MDA-MB

436/shControl or MDA-MB436/shERα36 cells promoted by E2. Ethanol was used as a control. All cells were treated with E2 for five days and cell number was determined each day. Each point indicates the mean (±SEM) of the results from three experiments. * $P < 0.05$.

C. The effects of tamoxifen and E2 on the growth of orthotopical xenograft tumors formed by MCF-7/mock cells.* $P < 0.05$.

D. Represent images of invasiveness of MCF-7/ERα36 or mock cells after treatment with E2 (1nM) or 4-OHT (1 μM) in a Transwell assay.

E. Decreased invasiveness of MDA-MB 436/shERα36 cells observed after E2 (1nM) or 4-OHT (1 μM) treatment in transwells. Each point indicates the mean (±SEM) of results from three experiments. * $p < 0.05$.

F. Representative H&E staining of metastases from 4T1-ERα$36^{+/-}$ cells in the mouse lung. BALB/c mice were orthotopically injected with 1×10^5 FACS-sorted 4T1-ERα$36^{+/-}$ cells, then were treated with *i.v.* injection tamoxifen (1 mg/kg) or E2 (1 nM/kg) through every three days for 18 days.

G. Metastatic foci in the lungs of mice analyzed with confocal microscopy, IHC and H&E staining. Mice xenografted with MCF-7/ERα36 cells via tail vein injection were treated with tamoxifen or E2. IHC staining of Fascin 1 was performed to confirm metastases in the lungs. Scale bar = 50μm.
