# Supplementary material for: Tamoxifen enhances stemness and promotes metastasis of ERα36+ breast cancer by upregulating ALDH1A1 in cancer cells
Source: Cell Res. 2018 Feb 2;28(3):336–58. doi: 10.1038/cr.2018.15 (PMC5835774; doi:10.1038/cr.2018.15)
Supplement: Supplementary information, Figure S5 — ERα36 maintains breast cancer cell stemness [file cr201815x5.pdf]

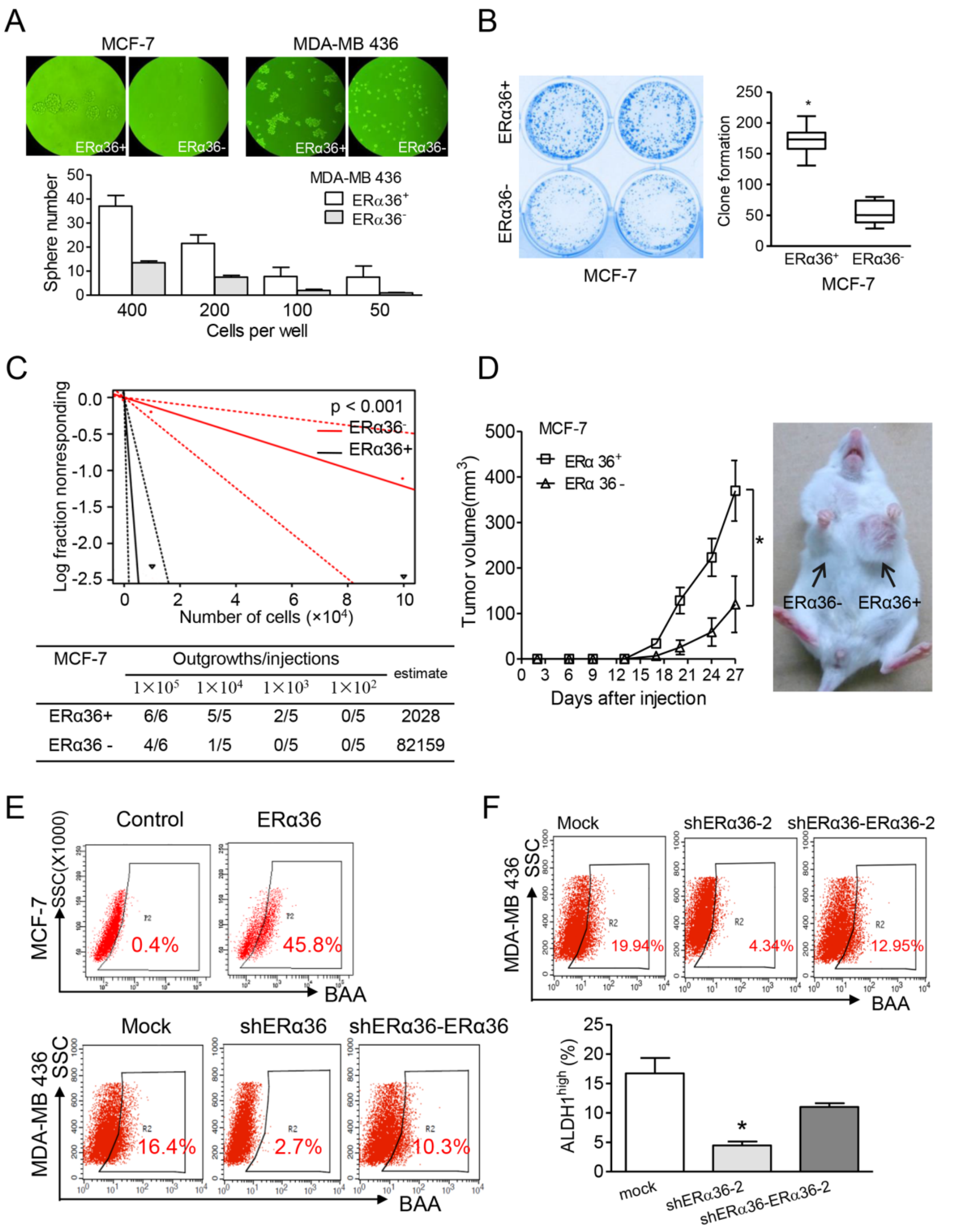

MCF-7	Outgrowths/injections				estimate
	1×10^5	1×10^4	1×10^3	1×10^2	
ERα36+	6/6	5/5	2/5	0/5	2028
ERα36 -	4/6	1/5	0/5	0/5	82159

Wang Q, *et al*. Figure S5

Figure S5. ERα36 maintains breast cancer cell stemness

A. Representative mammosphere formation images of ERα36$^{+/-}$ cells from MCF-7 and MDA-MB 436. MDA-MB 436-ERα36$^{+/-}$ cells were cultured with DF12 medium for 2 weeks for mammosphere formation. Number of spheres was presented as the

mean of spheres formed by indicated number of cells seeded; n =4. * $p < 0.01$.

B. Increased colonies observed with MCF-7-ERα36^{+} cells. Data were presented as the mean ± SEM. n = 6. * $p < 0.01$.

C. Limiting dilution analysis showing higher tumorigenic efficiency of FACS-sorted ERα36^{+} cells of MCF-7 cell line in NOD/SCID mice (n = 7). Black line refers to ERα36^{+} cells, red line for ERα36^{-} cells.

D. More rapid growth of orthotopical xenograft tumors formed by FACS-sorted MCF-7-ERα36^{+} cells (n = 5). The volume of xenograft tumors was measured at indicated time points. Data are presented as the means ± SEM. * $p < 0.01$.

E. Flow cytometry showing higher percentage of ALDH1^{high} cells in ERα36^{+} cell variants (MCF-7/ERα36, MDA-MB 436/shERα36-ERα36). n = 4.

F. Flow cytometry showing lower percentage of ALDH1^{high} cells in MDA-MB 436/shERα36(2). n = 3. * $p < 0.01$.
