# Supplementary material for: Tamoxifen enhances stemness and promotes metastasis of ERα36+ breast cancer by upregulating ALDH1A1 in cancer cells
Source: Cell Res. 2018 Feb 2;28(3):336–58. doi: 10.1038/cr.2018.15 (PMC5835774; doi:10.1038/cr.2018.15)
Supplement: Supplementary information, Figure S6 — Association of increased ALDH1A1 expression with poor prognosis in breast cancer patients. [file cr201815x6.pdf]

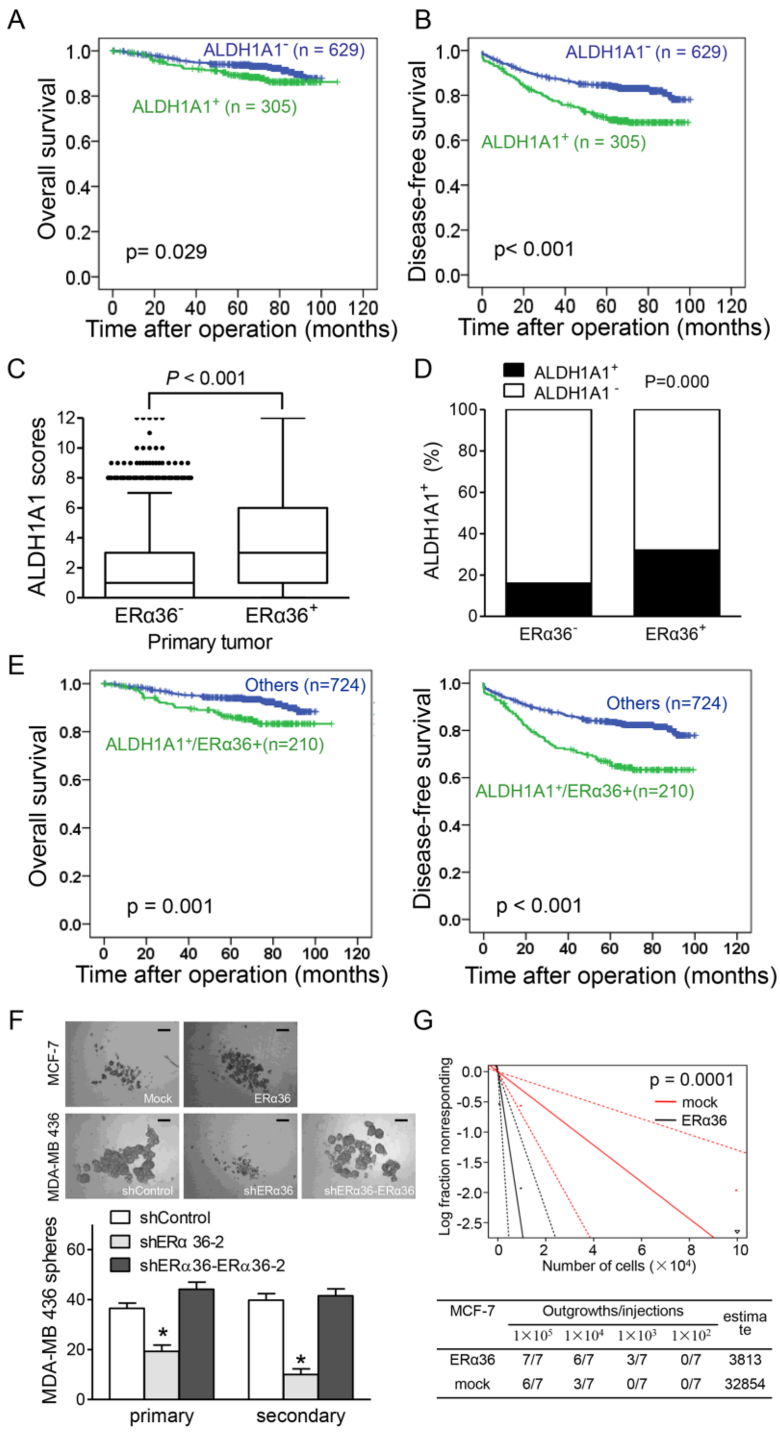


Wang Q, *et al*. Figure S6

**Figure S6. Association of increased ALDH1A1 expression with poor prognosis in breast cancer patients.**

A-B. Kaplan-Meier estimation of OS (A) and DFS (B) of breast cancer patients in Chongqing Cohort with ALDH1A1$^{+}$ or ALDH1A1$^{-}$ tumors. Worse prognosis was

observed in patients with ALDH1A1$^+$ tumors. Comparison was made between ALDH1A1$^+$ (score $\geq 5$) and ALDH1A1$^-$ (score $< 5$) patients.

C. Higher IHC scores of ALDH1A1 shown in ERα36$^+$ tumors. Data were analyzed using one-way analysis of variance (ANOVA) test with Games-Howell's correction.

D. Higher percentage of ALDH1A1$^+$ breast cancer samples in patients with ERα36$^+$ than ERα36$^-$ tumors. Data were analyzed using Pearson's $\chi 2$ test.

E. Kaplan-Meier estimation of OS and DFS of breast cancer patients in Chongqing Cohort with a combination of ALDHA1A1$^+$/ERα36$^+$ expression, which suggests a worse prognosis of ALDHA1A1$^+$/ERα36$^+$ patients than others.

F. Mammosphere number formed by primary and secondary generations of infected MDA-MB 436 cells. Reduced mammospheres were formed by MDA-MB 436/shERα36(2) cells. n = 3. Scale bar = 50 μm. * $p < 0.05$.

G. Limiting dilution showing increased tumorigenicity of MCF-7/ERα36 cells in NOD/SCID mice (7 mice each group).
