# Supplementary material for: Tamoxifen enhances stemness and promotes metastasis of ERα36+ breast cancer by upregulating ALDH1A1 in cancer cells
Source: Cell Res. 2018 Feb 2;28(3):336–58. doi: 10.1038/cr.2018.15 (PMC5835774; doi:10.1038/cr.2018.15)
Supplement: Supplementary information, Figure S7 — Enhanced stemness of ERa36+ cells by tamoxifen treatment. [file cr201815x7.pdf]

A

Gene Symbol	Fold regulation (log2)	GO Biological Process Term
TGFB2	4.0806	cell morphogenesis, angiogenesis
CLDN1	3.9446	cell adhesion, calcium-independent cell-cell adhesion
CYP1A1	3.70453	response to hypoxia, porphyrin-containing compound metabolic process
ITGB6	3.27408	inflammatory response, cell adhesion
PLEKHS1	2.94623	---
FAM5B	-3.1499	nervous system development, negative regulation of cell cycle
IGFBP5	-3.2972	regulation of cell growth
UGT2B15	-3.855	xenobiotic metabolic process, steroid metabolic process
UGT2B15	-3.855	xenobiotic metabolic process, steroid metabolic process
UGT2B7	-4.4751	lipid metabolic process, cellular glucuronidation

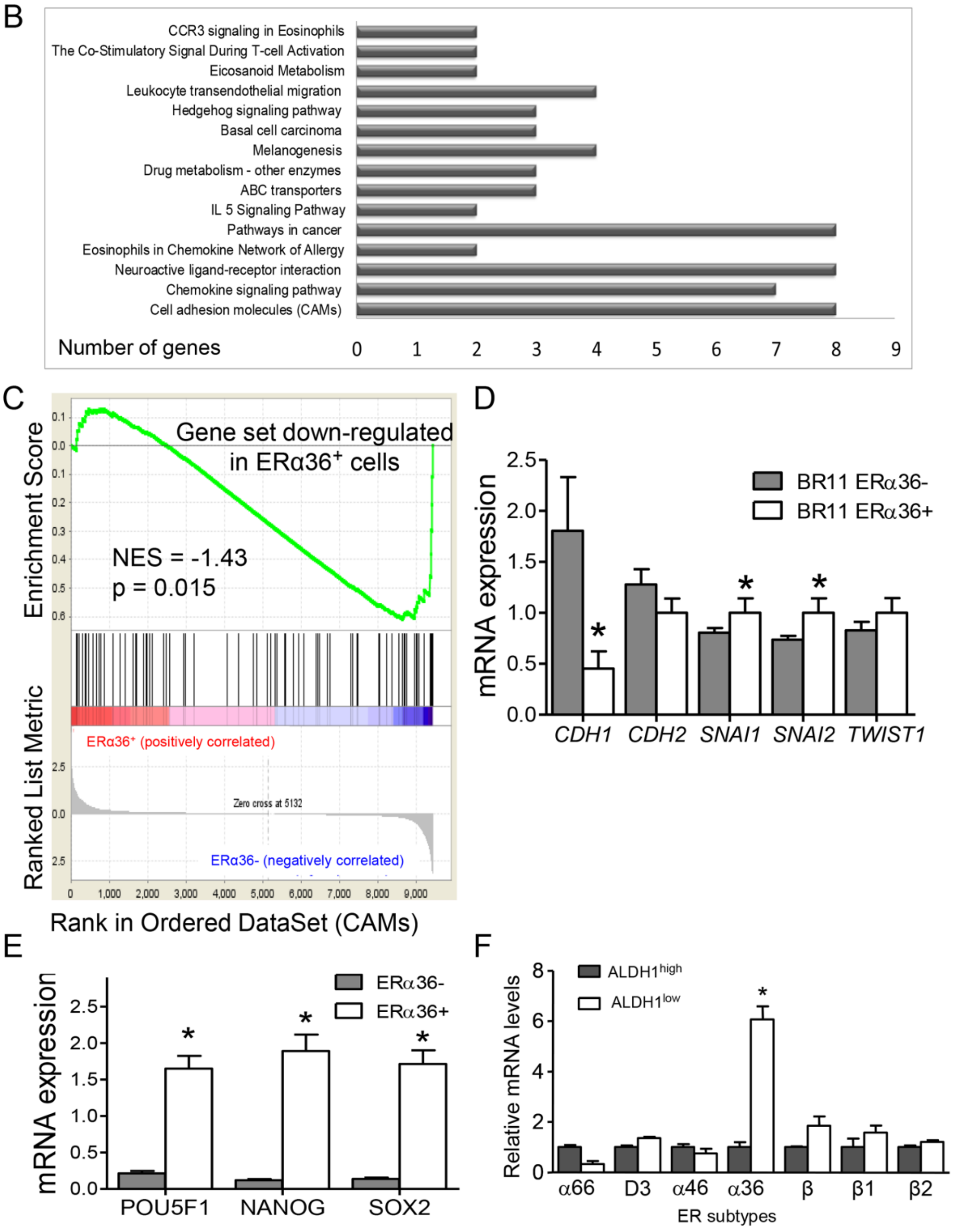

Wang Q, *et al*. Figure S7

Figure S7. Enhanced stemness of ERa36⁺ cells by tamoxifen treatment.

A. Ten most highly up-regulated or down-regulated genes in FACS sorted MCF-7-ERα36$^+$ cells pretreated with 4-OHT (1 μM).

B. The most different signaling pathways were compared between FACS sorted MCF-7-ERα36$^{+/-}$ cells.

C. Detection of genes coding for cell adhesion molecules in FACS sorted MCF-7-ERα36$^+$ cells treated with 4-OHT. NES denotes normalized enrichment score in gene-set enrichment analysis.

D. mRNA levels of CDH1/2, SNAI1/2 and TWIST1 in FACS sorted BR11-ERα36$^+$ cells and ERα36$^-$ cells determined by quantitative real-time RT-PCR (* $p < 0.01$).

E. mRNA levels of POU5F1, SOX2 and NANOG in FACS-sorted BR11-ERα36$^+$ and ERα36$^-$cells measured by quantitative real-time RT-PCR. Significant higher levels of embryonic stem cell markers in ERα36$^+$ cells were observed (*, $p < 0.01$).

F. Higher level of ERα36 mRNA detected in FACS-sorted MCF-7-ALDH1^{high} cells. * $p < 0.01$.
