# Supplementary material for: Tamoxifen enhances stemness and promotes metastasis of ERα36+ breast cancer by upregulating ALDH1A1 in cancer cells
Source: Cell Res. 2018 Feb 2;28(3):336–58. doi: 10.1038/cr.2018.15 (PMC5835774; doi:10.1038/cr.2018.15)
Supplement: Supplementary information, Figure S8 — Requirement of ERα36 for the induction of ALDH1A1 expression in breast cancer cells treated with estrogen or tamoxifen [file cr201815x8.pdf]

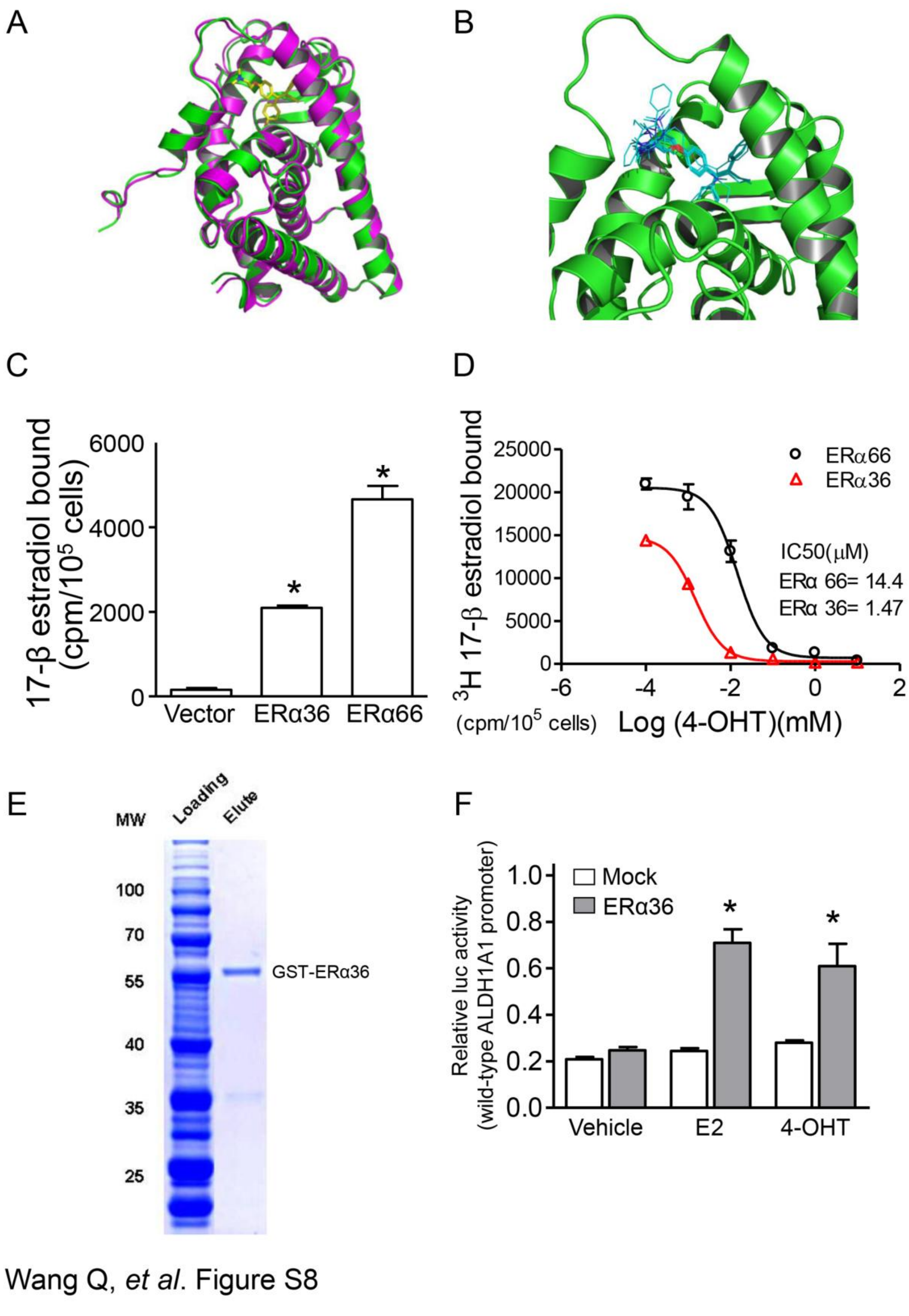

Figure S8. Requirement of ERα36 for the induction of ALDH1A1 expression in breast cancer cells treated with estrogen or tamoxifen

A. Three-dimensional structures of ERα36 and ERα66. Crystal composite structure in

three-dimensional model of the ligand binding domain of ERα36 (green) and ERα66 (purple) were shown, 4-OHT was represented by a stick.

B. Overall three-dimensional structure of ERα36 and docking with 4-OHT, which was analyzed with CDOCKER based on semi-flexible molecular docking program of CHARM force field.

C. ^{3}H-17-β estradiol binding to ERs transfected cells. * $p < 0.05$.

D. Competition of ^{3}H-17-β estradiol binding to ERα36 and ERα66 transfected cells by unlabeled 4-OHT. IC50 values were estimated by fitting the data to a nonlinear four-parameter logistic model. Results are representative of three experiments performed in duplicate samples.

E. Purified GST-ERα36 protein shown by coomassie brilliant blue staining. A major band of GST-ERα36 was observed with a lower minor band characterized as a premature N-terminal fragment of ERα36.

F. Luciferase activity of the reporter fused to a wild-type *ALDH1A1* promoter in MDA-MB 436/shERα36 and MDA-MB 436/shERα36-ERα36cells treated with E2 (1 nM) or 4-OHT (0.1 μM). * $p < 0.05$.
