# Supplementary material for: Tamoxifen enhances stemness and promotes metastasis of ERα36+ breast cancer by upregulating ALDH1A1 in cancer cells
Source: Cell Res. 2018 Feb 2;28(3):336–58. doi: 10.1038/cr.2018.15 (PMC5835774; doi:10.1038/cr.2018.15)
Supplement: Supplementary information, Figure S9 — Reduced self-renewal ability of ERα36+ cells by ALDH1A1 knockdown [file cr201815x9.pdf]

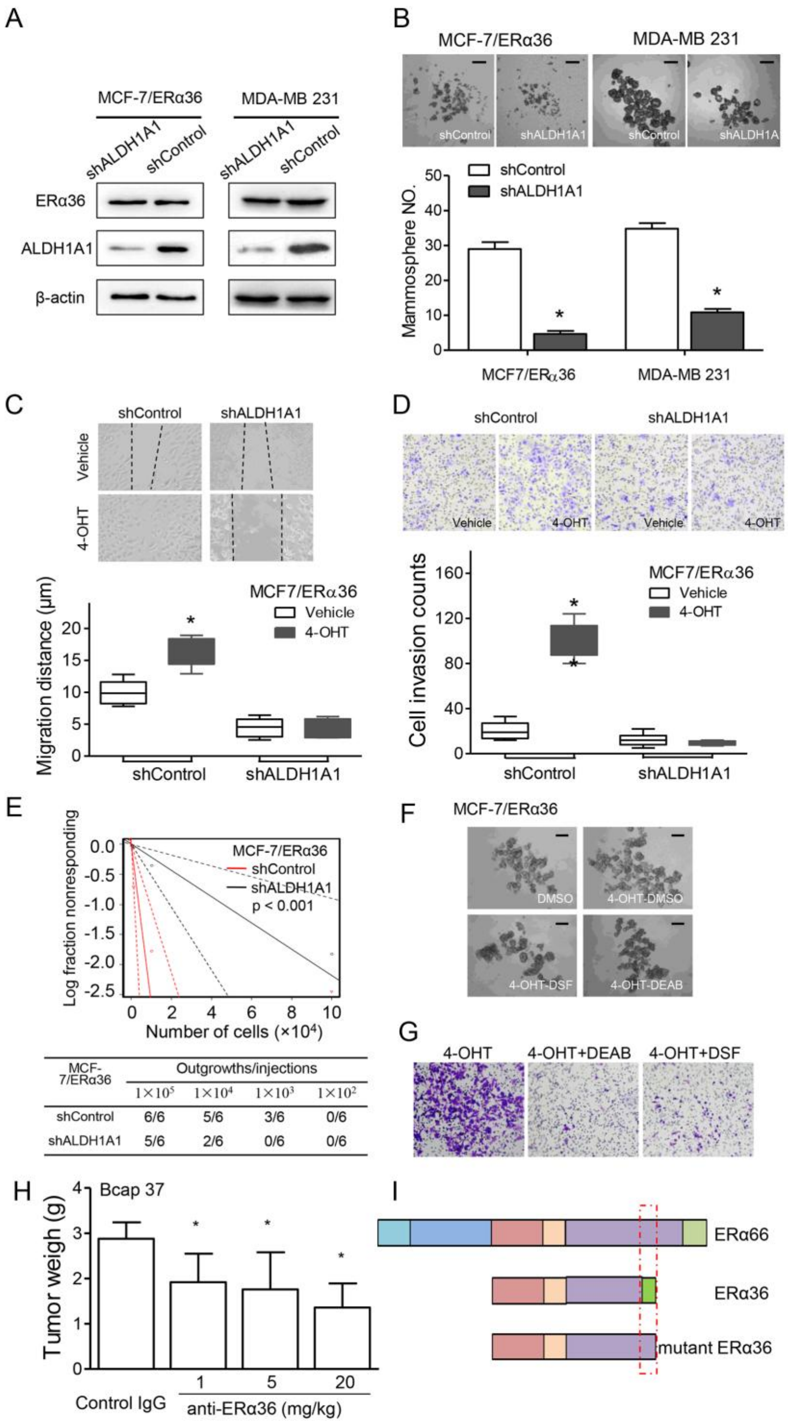

Wang Q, *et al*. Figure S9

Figure S9. Reduced self-renewal ability of ERα36^{+} cells by *ALDH1A1* knockdown

A. Immunoblotting of ALDH1A1 and ERα36 in MCF-7/ERα36 and MDA-MB 231 with *ALDH1A1* knockdown (-shALDH1A1 or -shControl cells). β-actin was used as

loading control.

B. Reduced mammosphere formation of MCF7/ERα36 and MDA-MB 231 cells in the presence of 4-OHT (1 μM) by *ALDH1A1* knockdown. Representative images were shown. Data were shown as the mean ± SEM. Scale bar = 50 μm. * $p < 0.05$.

C. *ALDH1A1* knockdown attenuates cell invasiveness of MCF-7/ERα36 cells with 4-OHT treatment (1 μM) in transwell. DMSO was used as a vehicle control. Representative images were shown. n = 3. * $p < 0.05$.

D. Reduced invasion ability observed in MCF-7/ERα36-shALDH1A1 cells with 4-OHT (1 μM) treatment in transwell. DMSO was used as a vehicle control. Representative images were shown. Each point indicates the mean ± SEM of results. * $p < 0.05$.

E. Significant reduction of tumorigenicity of MCF-7/ERα36 cells with *ALDH1A1* knockdown in the presence of tamoxifen with limiting dilution analysis (n = 7).

F. Reduction of mammosphere formation by MCF7/ERα36 cells in the presence of 4-OHT (1 μM) by ALDH1 inhibitors, diethylaminobenzaldehyde (DEAB, 10 nM) or Disulfiram (DSF, 0.1 μM). DMSO was used as a vehicle control. Representative images were shown. Scale bar = 50 μm.

G. Attenuation of the invasive ability of MDA-MB 436 cells after 4-OHT treatment (1 μM) in transwell assays by ALDH1 inhibitors DEAB and DSF. Representative images were shown.

H. Growth inhibition of xenografted tumors in the presence of tamoxifen by a monoclonal anti-ERα36 antibody. Decreased tumor weight was observed after ERα36

antibody treatment. NOD/SCID mice (7/group) were orthotopically injected with human Bcap-37 cells (1 x 10^6). When tumor size reached 200 mm^3, an anti-ERα36 monoclonal antibody (20 mg/ kg body weight) was administered through the tail vein every 3 or 4 days. An irrelevant IgG was injected as a control.

I. ERα36-Δ plasmid was prepared with a mutant of aa285 to 310. The sequence of ERα66 from aa 456 to 481 was used in the mutant ERα36.
