# Supplementary material for: Tamoxifen enhances stemness and promotes metastasis of ERα36+ breast cancer by upregulating ALDH1A1 in cancer cells
Source: Cell Res. 2018 Feb 2;28(3):336–58. doi: 10.1038/cr.2018.15 (PMC5835774; doi:10.1038/cr.2018.15)
Supplement: Supplementary information, Table S1 — The Correlation between ERα36 Expression and ClinicopathologicalCharacteristics in Breast Cancer Patients from Cohort Chongqing (n=1 068 cases) [file cr201815x10.pdf]

Supplementary Tables

Table S1. The Correlation between ERα36 Expression and Clinicopathological Characteristics in Breast Cancer Patients from Cohort Chongqing (n=1 068 cases)

Characteristic	Number (%)	ERα36 expression		p value
Total	1068	Positive	Negative	
		(493, 46.2%)	(575, 53.8%)	
Age, years				
<50	644 (60.3%)	294 (27.5%)	350 (32.8%)	0.681
≥50	424 (39.7%)	199 (18.6%)	225 (21.1%)	
Tumor size, cm				
≤2	292 (27.3%)	119 (11.1%)	173 (16.2%)	
2~5	645 (60.4%)	301 (28.2%)	344 (32.2%)	<0.001
≥5	131 (12.3%)	73 (6.8%)	58 (5.4%)	
Histological grades				
I	349 (32.7%)	72 (6.7%)	277 (25.9%)	
II	594 (55.6%)	354 (33.1%)	240 (22.5%)	<0.001
III	125 (11.7%)	67(6.3%)	58 (5.4%)	
Lymph node status				
0	447 (41.9%)	137 (12.8%)	310 (29.0%)	
1-3	267 (25.0%)	159 (14.9%)	108 (10.1%)	<0.001
≥4	203 (19.0%)	134 (12.5%)	69 (6.5%)	
Unknown	151 (14.1%)	63 (5.9%)	88 (8.2%)	

ERα66				
Positive	734 (68.7%)	329 (30.8%)	405 (37.9%)	0.193
Negative	334 (31.3%)	164 (15.4%)	170 (15.9%)	
PR				
Positive	637 (59.6%)	276 (25.8%)	361 (33.8%)	0.024
Negative	431 (40.4%)	217 (20.3%)	214 (20.0%)	
HER2				
Positive	118 (11.0%)	62 (5.8%)	56 (5.2%)	0.140
Negative	950 (89.0%)	431 (40.4%)	519(48.6%)	
Adjuvant therapy				
Chemotherapy	515 (48.2%)	230 (21.5%)	285 (26.7%)	
Chemotherapy	458 (42.9%)	228 (21.3%)	230 (21.5%)	
+ Tamoxifen				0.099
Tamoxifen alone	26 (2.4%)	14 (1.3%)	12 (1.1%)	
No treatment	23 (2.2%)	16 (1.5%)	7 (0.7%)	
Unknown	15 (1.4%)	5 (0.5%)	10 (0.9%)	

Abbreviations: ERα36, estrogen receptor-α36; ERα66, estrogen receptor-α66; PR, progesterone receptor; HER-2, human epidermal growth factor receptor 2. Two-sides Chi-Square tests.
