# Supplementary material for: Tamoxifen enhances stemness and promotes metastasis of ERα36+ breast cancer by upregulating ALDH1A1 in cancer cells
Source: Cell Res. 2018 Feb 2;28(3):336–58. doi: 10.1038/cr.2018.15 (PMC5835774; doi:10.1038/cr.2018.15)
Supplement: Supplementary information, Table S2 — Prognosis of Patients with ERα36+ or ERα36-Breast Cancer in Four Independent Cohorts (n=609) [file cr201815x11.pdf]

Table S2. Prognosis of Patients with ERα36$^+$ or ERα36$^-$ Breast Cancer in Four Independent Cohorts (n = 609)

Cohorts	Case No.	Metastasis	p Value	Death	p Value
Beijing (I)					
ERα36$^+$	77	35	<0.001	18	0.008
ERα36$^-$	87	13		7	
Chengdu (II)					
ERα36$^+$	59	9	0.007	4	0.124
ERα36$^-$	92	2		2	
Guangzhou (III)					
ERα36$^+$	83	13	0.012	2	1.000
ERα36$^-$	71	2		1	
Chongqing II (IV)					
ERα36$^+$	70	15	0.001	3	0.245
ERα36$^-$	70	2		0	
Total					
ERα36$^+$	289	72	<0.001	27	0.002
ERα36$^-$	320	19		10	

Abbreviations: ERα36, estrogen receptor-α36; 2-sides Chi-Square Tests.
