# Supplementary material for: Tamoxifen enhances stemness and promotes metastasis of ERα36+ breast cancer by upregulating ALDH1A1 in cancer cells
Source: Cell Res. 2018 Feb 2;28(3):336–58. doi: 10.1038/cr.2018.15 (PMC5835774; doi:10.1038/cr.2018.15)
Supplement: Supplementary information, Table S3 — Multivariate Analyses of Disease-Free Survival (DFS) and Metastases-Free Survival (MSF) in 342 Patients with Breast Cancer Positive for Both ERα36 and ERα66 [file cr201815x12.pdf]

Table S3. Multivariate Analyses of Disease-Free Survival (DFS) and Metastases-Free Survival (MSF) in 342 Patients with Breast Cancer Positive for Both ERα36 and ERα66

Factors	Disease-Free Survival			Metastases-Free Survival		
	#HR	95%CI	p	HR	95%CI	p
Age	0.899	0.494 - 1.635	0.728	0.666	0.332 - 1.336	0.253
Size	1.512	0.724 - 3.157	0.271	1.966	0.756 - 5.112	0.166
*LNM	2.283	1.104 - 4.723	0.026	1.986	0.871 - 4.530	0.103
Stage	1.509	0.626 - 3.635	0.359	3.970	0.894 - 17.621	0.070
Grade	1.311	0.523 - 3.287	0.564	1.530	0.448 - 5.227	0.497
PR (+)	0.511	0.291 - .899	0.020	0.343	0.182 - 0.646	0.001
HER2 (+)	1.635	0.690 - 3.874	0.264	2.043	0.841 - 4.963	0.115
Tamoxifen	5.326	2.096 - 13.536	0.000	4.037	1.560 - 10.443	0.004

*LNM, Lymph node metastasis. #HR, Hazard ratio.
