# Supplementary material for: Tamoxifen enhances stemness and promotes metastasis of ERα36+ breast cancer by upregulating ALDH1A1 in cancer cells
Source: Cell Res. 2018 Feb 2;28(3):336–58. doi: 10.1038/cr.2018.15 (PMC5835774; doi:10.1038/cr.2018.15)
Supplement: Supplementary information, Table S4 — Responses of Tamoxifen Treatment to Patients with ERα36+ or ERα36− Breast Cancer in Four Independent Cohorts [file cr201815x13.pdf]

Table S4. Responses of Tamoxifen Treatment to Patients with ERα36^{+} or ERα36^{-} Breast Cancer in Four Independent Cohorts

Cohorts	No. of Patients	Metastasis	p value	Death	p value
Beijing (I)					
ERα36^{+}	35	10	<0.001	2	<0.001
ERα36^{-}	53	0		0	
Chengdu (II)					
ERα36^{+}	35	8	<0.001	4	0.239
ERα36^{-}	45	2		2	
Guangzhou (III)					
ERα36^{+}	45	12	0.005	2	1.000
ERα36^{-}	38	1		1	
Chongqing II (IV)					
ERα36^{+}	55	14	0.269	3	1.000
ERα36^{-}	13	1		0	
Total					
ERα36^{+}	170	44	<0.001	11	0.026
ERα36^{-}	149	4		3	

Abbreviations: ERα36, estrogen receptor-α36; 2-sides Chi-Square tests.
