# Supplementary material for: Tamoxifen enhances stemness and promotes metastasis of ERα36+ breast cancer by upregulating ALDH1A1 in cancer cells
Source: Cell Res. 2018 Feb 2;28(3):336–58. doi: 10.1038/cr.2018.15 (PMC5835774; doi:10.1038/cr.2018.15)
Supplement: Supplementary information, Table S5 — Responses of Patients with ERα36+ Breast Cancer to Tamoxifen or Others in Four Cohorts [file cr201815x14.pdf]

Table S5. Responses of Patients with ERα36$^+$ Breast Cancer to Tamoxifen or Others in Four Cohorts

Cohorts	No. of Patients	Metastasis	p value	Death	p value
Beijing (I)					
Tamoxifen	35	10	0.005	2	0.523
Others	21	4		0	
Chengdu (II)					
Tamoxifen	33	8	0.009	4	0.077
Others	24	0		0	
Guangzhou (III)					
Tamoxifen	45	12	0.005	2	0.499
Others	37	1		0	
Chongqing II (IV)					
ERα36$^+$	55	14	0.005	3	0.247
ERα36$^-$	47	2		0	
Total					
Tamoxifen	168	44	<0.001	11	0.003
Others	129	7		0	
