# Supplementary material for: Tamoxifen enhances stemness and promotes metastasis of ERα36+ breast cancer by upregulating ALDH1A1 in cancer cells
Source: Cell Res. 2018 Feb 2;28(3):336–58. doi: 10.1038/cr.2018.15 (PMC5835774; doi:10.1038/cr.2018.15)
Supplement: Supplementary information, Table S6 — Postmenopausal Breast Cancer Patient Groups Treated with Aromatase Inhibitors (AIs) and/or Tamoxifen with Tumors Expressing both ERα36 and ERα66 (n=244) [file cr201815x15.pdf]

Table S6. Postmenopausal Breast Cancer Patient Groups Treated with Aromatase Inhibitors (AIs) and/or Tamoxifen with Tumors Expressing both ERα36 and ERα66 (n = 244)

	With tamoxifen	With AIs	P
TE	102	89	
CEF	22	22	0.816
Other	4	5	
Total	128	116	

Abbreviations: AIs: Aromatase inhibitors; TE: Docetaxel and epirubicin; CEF: Cyclophosphamide, epirubicin, and fluorouracil.
