# Supplementary material for: Tamoxifen enhances stemness and promotes metastasis of ERα36+ breast cancer by upregulating ALDH1A1 in cancer cells
Source: Cell Res. 2018 Feb 2;28(3):336–58. doi: 10.1038/cr.2018.15 (PMC5835774; doi:10.1038/cr.2018.15)
Supplement: Supplementary information, Table S7 — Multivariate Analyses of Disease-Free Survival (DSF) and Metastasis-Free Survival (MSF) of Postmenopausal Patients with ERα36+/ERα66+ breast cancer [file cr201815x16.pdf]

Table S7. Multivariate Analyses of Disease-Free Survival (DSF) and Metastasis-Free Survival (MSF) of Postmenopausal Patients with ERα36^{+}/ERα66^{+} breast cancer

Factor	Disease-Free Survival			Metastases-Free Survival		
	HR	95%CI	p	HR	95%CI	P
Size	2.623	0.579 - 11.876	0.211	3.279	0.414 – 25.976	0.261
LNM	5.858	1.194 - 28.738	0.029	6.951	0.827 – 58.428	0.074
Stage	9.977	0.891 - 111.713	0.062	27569	0.000 - 144700	0.957
Grade	0.215	0.032 - 1.421	0.111	0.255	0.025 - 2.569	0.246
PR (+)	1.840	0.593 - 5.708	0.291	1.292	0.360 - 4.643	0.695
HER2 (+)	1.327	0.152 - 11.569	0.798	1.649	0.174 – 15.599	0.663
Tamoxifen	7.705	1.699 - 34.944	0.008	4.098	0.859 – 19.539	0.077
AIs	0.779	0.282 - 2.147	0.629	0.664	0.197 - 2.238	0.509

Abbreviations: ERα36, estrogen receptor-α36; ERα66, estrogen receptor-α66; HR: hazard ratio; Stage: clinical stage; Grade: histological grade； LNM: Lymph node metastasis; PR (+): progesterone receptor-positive; HER2 (+), human epidermal growth factor receptor 2-positive; Tamoxifen: tamoxifen treatment; AIs: aromatase inhibitors treatment.
